# The Role of the Gut Microbiota in Modulating Signaling Pathways and Oxidative Stress in Glioma Therapies

**DOI:** 10.3390/cancers17050719

**Published:** 2025-02-20

**Authors:** Aleksandra Krawczyk, Gabriela Elzbieta Sladowska, Barbara Strzalka-Mrozik

**Affiliations:** Department of Molecular Biology, Faculty of Pharmaceutical Sciences in Sosnowiec, Medical University of Silesia, 40-055 Katowice, Poland; s81894@365.sum.edu.pl (A.K.); s81949@365.sum.edu.pl (G.E.S.)

**Keywords:** glioma, microbiota–gut–brain axis, signaling pathways, oxidative stress, immunotherapy

## Abstract

Tumors of the central nervous system (CNS), especially gliomas, pose a significant clinical challenge due to their aggressive nature and limited therapeutic options. Emerging research highlights the critical role of the gut microbiota in regulating CNS health and disease. The composition of the gut microbiota is essential for maintaining CNS homeostasis, as it modulates immune responses, oxidative status, and neuroinflammation. The microbiota–gut–brain axis, a bidirectional communication network, plays a pivotal role in cancer and CNS disease treatment, exerting its influence through neural, endocrine, immunological, and metabolic pathways. Recent studies suggest that the gut microbiota influences the solidification of the tumor microenvironment and that dysbiosis may promote glioma development by modulating systemic inflammation and oxidative stress, which contributes to tumorigenesis and CNS tumor progression. This review interrogates the impact of the gut microbiota on glioma, focusing on critical pathways such as NF-κB, MAPK, PI3K/Akt/mTOR, and Kynurenine/AhR that drive tumor proliferation, immune evasion, and therapy resistance. Furthermore, we explore emerging therapeutic strategies, including probiotics and microbiota-based interventions, which show potential in modulating these pathways and enhancing immunotherapies such as checkpoint inhibitors. By focusing on the multifaceted interactions between the gut microbiota, oxidative stress, and CNS tumors, this review highlights the potential of microbiota-targeted therapies and their manipulation to complement and enhance current treatments.

## Simple Summary

Gliomas are aggressive brain tumors with limited treatment options and poor survival rates. Recent research suggests that the gut microbiome plays a crucial role in tumor growth and therapy response by interacting with key cellular pathways involved in inflammation, cell survival, and oxidative stress. This study investigates the impact of gut microbiota on immune responses and tumor progression through critical signaling pathways. Our goal is to identify potential strategies to enhance glioma treatments by targeting gut bacteria. Gaining a deeper understanding of these interactions could provide novel insights into how gut microbiota may be leveraged to support cancer therapy.

## 1. Introduction

Tumors of the central nervous system (CNS), particularly gliomas, remain among the most aggressive and treatment-resistant malignancies, posing a major challenge to modern oncology. These are rare and heterogeneous cancers, and predictions about the potential course of the disease vary depending on the age of the patient and the tumor’s histological type [1]. Despite advancements in surgical techniques, radiotherapy, and pharmacological interventions, the prognosis for patients with CNS tumors remains poor, highlighting the urgent need for innovative therapeutic strategies. Emerging evidence indicates that the gut microbiota serves as a critical modulator of CNS health and disease, offering novel insights into the pathophysiology of CNS tumors and potential therapeutic avenues [1].

The gut microbiota plays a critical role in maintaining CNS homeostasis through the microbiota–gut–brain axis, which is a complex communication network that influences neural, endocrine, immune, and metabolic pathways [2,3]. The microbiota produces metabolites [4,5,6] and neurotransmitters that interact with gut nerve receptors and travel to the brain, affecting processes such as mood, memory, and stress responses [7,8,9,10,11,12].

Moreover, key signaling pathways commonly associated with cancer progression, such as the nuclear factor kappa B (NF-κB), mitogen-activated protein kinase (MAPK), phosphoinositide 3-kinase (PI3K)/protein kinase B (Akt)/mammalian target of rapamycin (mTOR), and kynurenine/aryl hydrocarbon receptor (AhR) pathways, are influenced by the gut microbiota, highlighting a potential link between microbial composition and cancer biology [13].

Understanding the mechanisms of these pathways is essential to identifying novel therapeutic targets and opportunities for modulating the microbiota in the treatment of CNS cancers. Dysbiosis of the gut microbiota has been associated with various neurological conditions, including CNS cancers, by promoting systemic inflammation, oxidative stress, and immune dysregulation [14,15].

This review aims to explore the multifaceted role of the gut microbiota in CNS tumors, focusing on its influence on oxidative stress, signaling pathways, and immune responses. Additionally, the potential of microbiota-targeted therapies, including their integration with advanced immunotherapies, is discussed as a promising approach to improve outcomes in CNS malignancies. Gaining insight into these interactions may pave the way for innovative therapeutic strategies that address the complex interplay between the gut microbiota and CNS cancers.

## 2. CNS Cancers

Tumors located in the nervous system represent a diverse group of diseases that vary in morphology, affect all age groups, and occur more frequently in men than in women [16]. CNS cancers rank as the 12th leading cause of cancer-related deaths worldwide (data for 2022) [17]. These rare and heterogeneous malignancies have highly variable prognoses, influenced by factors such as the patient’s age and the histological type of the tumor [1].

In 2022, the global incidence of primary malignancies in the brain and other central nervous system sites, adjusted for age demographics, was estimated at 3.5 cases per 100,000 individuals. Among children aged 0–14 years, pilocytic astrocytoma was the most common histopathological type, with an incidence of 1.25 cases per 100,000. In the 15–39 age group, pituitary tumors were predominant, with an incidence of 4.47 cases per 100,000, while glioblastoma multiforme (GBM) was the most frequent type in individuals aged 40 and older, showing an incidence of 21.88 cases per 100,000. These data were sourced from the Central Brain Tumor Registry of the United States (CBTRUS) [18].

The majority of cases involve the brain, but significant differences in the reported incidence and mortality rates of this type of cancer likely reflect variations in diagnostic capabilities and treatment access. In most countries, an upward trend in brain cancer morbidity and mortality has been observed. This increase, particularly in Western countries, is thought to be associated with advancements in diagnostic techniques. Data on the causes of brain tumors remain limited, although genetic factors and exposure to ionizing radiation have been identified as important contributors [1,19,20].

Gliomas are the most common histological type of brain tumors and represent the most frequent malignant primary brain tumors in adults. They can be categorized into limited and diffuse gliomas: the limited type is benign and treatable, while the diffuse type is malignant and cannot be effectively treated with surgical intervention alone.

In addition to gliomas, other tumors of glial origin include ependymomas and schwannomas. CNS tumors also encompass medulloblastomas, CNS lymphomas, and meningiomas [19,21].

Glioblastoma multiforme (GBM) arises through genetic and epigenetic alterations of normal cells, leading to the activation of proto-oncogenes, which drive tumorigenesis, and the inhibition of tumor suppressor genes that normally counteract tumor transformation. Multiple processes contribute to tumor formation, including loss of cell cycle control, disruption of apoptosis, and genetic instability [22].

Diagnostic tools for GBM include magnetic resonance imaging (MRI) with contrast, computed tomography (CT), and positron emission tomography (PET), among others. Treatment typically involves a combination of surgical intervention, radiation therapy, and chemotherapy, tailored to the specific type and characteristics of the tumor [23].

## 3. Composition of Intestinal Microbiota Important in Maintaining CNS Homeostasis

The digestive system harbors the largest population of microorganisms in the human body, amounting to up to 2 kg of microbes, including bacteria, viruses, fungi, and protozoa. The number and composition of the intestinal microbial population are influenced by factors such as diet, medication use, body weight, ethnicity, and metabolic status. The predominant microbial genera in the gut include *Bacteroidetes* (20–25%), *Firmicutes* (60–65%), *Proteobacteria* (5–10%), and *Actinobacteria* (3%) (Table 1) [24,25,26,27,28,29,30,31,32].

A growing body of research suggests that the gut microbiota may influence brain function [38,39,40]. Findings from a study by Diaz et al. [38] demonstrated that microbial colonization plays a role in initiating signaling mechanisms that affect neuronal circuits responsible for motor- and anxiety-related behaviors. Compared to specific pathogen-free (SPF) mice with a normal gut microbiota, germ-free (GF) mice exhibited increased motor activity and reduced anxiety levels. Additionally, the presence of gut microbiota in GF mice was associated with a decreased expression of proteins involved in synaptic maturation, such as synaptophysin and PSD-95 [38].

Gut microbes are also known to regulate the development of the nervous system, which governs intestinal motility and transmits signals to the CNS [30,31].

The cell walls of certain Gram-negative bacteria contain lipopolysaccharides (LPSs), which play an important role in CNS function. LPSs can be transported from the intestinal mucosa into the systemic circulation, where they interact with toll-like receptors, triggering an immune response. This interaction leads to the activation of systemic inflammation and the production of pro-inflammatory cytokines, such as tumor necrosis factor-α (TNF-α), interleukin-6 (IL-6), and interleukin-1β (IL-1β) [30,31].

Additionally, bacteria belonging to the genus *Lactobacillus* are believed to secrete chemicals that inhibit the colonization of inflammatory microorganisms, potentially mitigating inflammatory responses [31].

Wikoff et al. [4] demonstrated that gut microbiota influence the levels and types of tryptophan metabolites in mammalian blood serum. Certain intestinal bacteria have the ability to produce tryptophanase, an enzyme that converts tryptophan into indole, pyruvate, and ammonia. Indole plays a critical role in strengthening the intestinal barrier and inducing the expression of genes involved in mucin production, thereby enhancing resistance to inflammation [4].

Exposure of inflammatory cells to indole can reduce inflammation by repressing NF-κB activation and decreasing the production of pro-inflammatory chemokines, while simultaneously increasing the production of anti-inflammatory cytokines. Systemic levels of tryptophan metabolites affect the concentration of neurotransmitters (e.g., serotonin and gamma-aminobutyric acid) in the CNS and influence the production of neurotoxic molecules in astrocytes and microglia [31,41].

Kabouridis et al. [42] showed that microorganisms and their products regulate the development and maturation of nervous system glial cells. The gut microbiota is essential for the proper development of the gut glial cell network (mEGC) in mice and is also essential for maintaining the homeostasis of these cells throughout life [42].

Disruptions in the organization and function of the intestinal glia—an important component of gut–brain axis communication—or deficits in CNS glial cells may contribute to neuroendocrine and behavioral abnormalities [30].

*Firmicutes* and *Bacteroidetes* are the primary producers of short-chain fatty acids (SCFAs), including butyrate, acetate, and propionate. Most SCFAs are generated through the anaerobic fermentation of dietary fiber by the host’s gut microbiota. These molecules are absorbed by intestinal epithelial cells via H+- or sodium-dependent monocarboxylate transporters. The unmetabolized portion of SCFAs serves as an energy substrate for hepatocytes, except for acetate, which is not oxidized in the liver [29,43,44,45].

Consequently, only a small amount of SCFAs reach the systemic circulation and peripheral tissues. Despite this, SCFAs significantly influence the CNS, affecting neurotransmitter metabolism, mitochondrial functionality, immune responses, and lipid metabolism. SCFAs also function as histone deacetylase inhibitors, promoting the acetylation of nucleosomal histones across various cell types and tissues, including the intestine, peripheral nervous system, and CNS. The production and type of SCFAs are strongly influenced by gut microbiota composition and dietary carbohydrate intake [29,43,44,45].

The composition and diversity of the intestinal microbiota can vary depending on the presence or absence of cancer, as well as its degree of malignancy. Patients with glioblastoma exhibited reduced microbiota diversity compared to controls, as well as a decreased *Firmicutes* to *Bacteroidetes* ratio [46]. Comparing patients with malignant and benign brain tumors, microbial diversity results were similar, but in the malignant tumor group, the abundance of Firmicutes was the lowest, whereas in the benign tumor group, Actinobacteria showed the lowest levels, in contrast to the control group [47]. Research is ongoing into the relationship between gut microbiota and glioblastoma multiforme (GBM) risk [48]. It has been shown that the family *Peptostreptococcaceae* and the genus *Eubacterium brachy* may be associated with a higher risk of GBM incidence, unlike the family *Ruminococcaceae* [49].

## 4. Microbiota–Gut–Brain Axis

A link exists between the gut microbiota and the central nervous system (CNS), particularly in regard to neurodegenerative diseases [50]. The entero-cerebral axis is a bidirectional communication channel connecting the intestines and the CNS, safeguarded by the blood–brain barrier (BBB) [51].

Evidence for the bidirectionality of this pathway is supported by studies such as those on irritable bowel syndrome (IBS), where stress exacerbates pain and digestive issues, demonstrating the brain’s influence on intestinal function. Conversely, the intestinal microbiota play a significant role in the gut–brain axis during eubiosis by reducing inflammation and enhancing the presence and availability of tryptophan—a pivotal precursor for serotonin and other metabolites, including kynurenine [52,53,54,55].

Under physiological conditions, the influence of the intestine on brain function is significant for maintaining mental well-being [39].

### 4.1. The Ways of Communication

**Figure 1:**
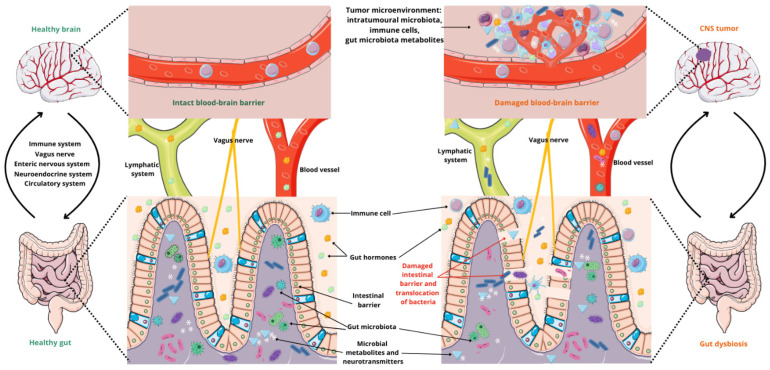
The gut–brain axis associated with CNS tumors. Parts of the figure were drawn by using pictures from Servier Medical Art, licensed under Creative Commons Attribution 4.0 International.

Communication between the gut microbiome, the intestines, and the brain is crucial to maintain synergy between the microbiota and the host. This interaction occurs through multiple pathways, including the autonomic nervous system, the immune system, the enteric nervous system (ENS), the neuroendocrine system, modulation of the hypothalamic–pituitary–adrenal (HPA) axis (associated with stress), the cardiovascular system, and microbial metabolites (Figure 1) [2,50,56,57].

One of the key communication pathways in the microbiota–gut–brain axis is through the vagus nerve, the primary component of the parasympathetic nervous system. It acts as a fundamental connection between the intestines and the brainstem, consisting of sensory and motor fibers that transmit information from visceral organs to the brain while also providing feedback to the viscera [2,58].

The afferent fibers of the vagus nerve do not penetrate the epithelial layer [59]. Instead, they detect microbiota signals of a mechanical, chemical, or hormonal nature through the diffusion of bacterial compounds, metabolites, or via gut endocrine cells. These signals are then transmitted to the nucleus of the solitary tract (NTS) in the brainstem, which relays the information to other brain structures, such as the hypothalamus and limbic system [56,58]. In turn, afferent fibers of the vagus nerve can activate efferent fibers through the inflammatory reflex, establishing the vagus nerve as a direct communication pathway between the brain and intestinal structures [58].

Studies on the effects of probiotics on the behavior of laboratory animals have demonstrated that administering specific probiotic strains to rats reduces cortisol secretion under stress. However, when the vagus nerve is severed, the effects of probiotics are entirely eliminated, underscoring the critical role of this pathway [39].

The enteric nervous system (ENS), often referred to as the “second brain”, is a complex network of neurons and glial cells responsible for regulating intestinal function and responding to metabolites produced by the gut microbiota [60,61]. Enteroendocrine cells (EECs) in the gut act as mediators between the intestinal lumen and the vagus nerve. These cells are activated by chemical stimuli, such as dietary components and bacterial metabolites (e.g., short-chain fatty acids (SCFAs) and tryptophan), which bind to their receptors. This activation induces the release of neurotransmitters like serotonin (5-HT) and hormones such as glucagon-like peptide 1 (GLP-1) and cholecystokinin (CCK) [56,58,62,63].

A key component of the immunological pathway within the gut–brain axis is the intestinal barrier, which prevents the translocation of pathogens and toxins into the bloodstream. This barrier is strengthened by tight intercellular junctions, regulated by cytokines and metabolites produced by the gut microbiota. The production of short-chain fatty acids (SCFAs), such as butyrate, by the microbiota is essential for maintaining the integrity of this barrier [56].

In cases of dysbiosis—an altered microbial balance—the gut barrier may become more permeable, allowing toxins and pathogens to enter the bloodstream. This increased permeability triggers immune responses in both the gut and the CNS. SCFAs, including butyrate and acetate, not only support intestinal barrier integrity but also modulate the activity of immunocompetent cells. These SCFAs can activate G-protein-coupled receptors (e.g., GPR43 and GPR41) expressed on immune cells, promoting the production of anti-inflammatory mediators and suppressing inflammation [25,56,58,64].

Neuroactive SCFAs have the unique ability to cross the blood–brain barrier via monocarboxylate transporters [65,66]. Once in the CNS, these SCFAs contribute to the maturation and function of microglial cells, further underscoring their critical role in gut–brain axis communication [67].

The microbiota produces various metabolites, including tryptophan derivatives, lipopolysaccharides (LPSs), peptidoglycans, bile acids such as deoxycholic acid (DCA), lithocholic acid, and ursodeoxycholic acid (UDCA), as well as trimethylamine N-oxide (TMAO) [4,5,6]. Additionally, it synthesizes neurotransmitters such as serotonin, gamma-aminobutyric acid (GABA), dopamine, norepinephrine, acetylcholine, and histamine. These compounds interact with gut nerve receptors and may migrate to the brain, influencing mood, memory, and stress responses [7,8,9,10,11,12].

Metabolites of serotonin, GABA, and tryptophan cannot cross the blood–brain barrier (BBB); instead, they indirectly affect the CNS by interacting with enteric nervous system (ENS) cells [68].

### 4.2. The Influence of Microbiota on Glioma Development

The gut microbiota exerts systemic effects on metabolism, cell proliferation, inflammation, and immunity, thereby potentially influencing cancer initiation, progression, and response to therapy [69]. Experiments conducted in mouse models have demonstrated that transferring the microbiota from tumor-bearing mice to tumor-free mice promotes carcinogenesis [70,71,72].

Emerging research further indicates that specific microbial communities and their metabolites can impact glioma progression and treatment responses, as summarized in Table 2.

*Bacteroides cellulosilyticus* is a member of the *Bacteroides* genus, a dominant group within the gut microbiota [108]. While direct evidence specifically linking *Bacteroides cellulosilyticus* to T cell activation remains limited, several members of the *Bacteroides* genus are well known for their ability to modulate immune responses, including T cell activation and differentiation [109,110].

*Bacteroides cellulosilyticus* WH2 possesses a large pool of carbohydrate-active enzymes, suggesting its capability to metabolize structurally diverse carbohydrates with varying degrees of polymerization [108,109]. Additionally, bacterial envelope polysaccharides from *B. cellulosilyticus* have been shown to influence immune regulation by affecting Treg and IL-10 activation in human peripheral blood mononuclear cells. This interaction has been linked to the stimulation of lymphocyte differentiation into Treg cells. However, it has been suggested that the observed changes in IL-10 levels should not be solely attributed to differences in Treg lymphocyte numbers, as other immune cells present in the blood may also contribute to interleukin production [110].

## 5. The Relationship Between Gut Microbiota, Oxidative Stress, and CNS Cancer

Nervous system cells, due to their high metabolic activity, produce significant amounts of reactive oxygen species (ROS). Excessive ROS production leads to oxidative stress, a harmful process that causes cellular damage [111]. Under physiological conditions, the REDOX balance maintains oxidative signaling while preventing damage. However, disruption of this balance—where ROS such as superoxide anion (O_2_^−^), hydrogen peroxide (H_2_O_2_), nitric oxide (NO) and hydroxyl radicals (·OH) exceed antioxidant levels—impairs normal function and disrupts intercellular signaling [112,113]. This imbalance contributes to the development of various diseases, including neurological disorders [111].

ROS are primarily produced by mitochondria and nicotinamide adenine dinucleotide phosphate (NADPH) oxidases. During mitochondrial oxidative phosphorylation, along with the production of ATP, superoxide anion (O_2_^−^) is generated, which is subsequently converted into hydrogen peroxide (H_2_O_2_) and molecular oxygen (O_2_). Mitochondrial ROS production is closely associated with the activity of the matrix enzyme aconitase, which converts H_2_O_2_ into hydroxyl radicals (·OH) through the Fenton reaction. The outer mitochondrial membrane also contributes to ROS generation via monoamine oxidases (MAOs). These enzymes catalyze the oxidative deamination of monoamines, with H_2_O_2_ produced as a by-product of the reaction [111].

Two isoforms of MAO are currently recognized. MAO-A is primarily located in catecholaminergic neurons, where it participates in the oxidation of neurotransmitters such as norepinephrine and serotonin. In contrast, MAO-B is predominantly expressed in serotonergic neurons and glial cells, where it oxidizes β-phenylethylamine [111].

NADPH oxidases catalyze the reduction of oxygen using NADPH as an electron donor [112]. Among these, NADPH oxidase 2 (NOX2) is particularly important and is located in phagocytes, neutrophils, B lymphocytes, and dendritic cells. NOX2 facilitates the transfer of electrons across the plasma membrane to extracellular oxygen, forming extracellular superoxide anion (O_2_^−^). This is subsequently converted into hydrogen peroxide (H_2_O_2_), which can diffuse back across the plasma membrane into the cell. In contrast, NADPH oxidase 1 (NOX1) is expressed in intestinal epithelial cells, where it can be induced by the intestinal microbiota. NOX1 plays critical roles in processes such as cell migration, differentiation, and wound healing [112].

When the gut microbial balance is disrupted, dysregulated bacteria can produce excessive ROS and impair intestinal barrier function. This disruption allows harmful substances and antigens to cross the intestinal lining, exacerbating oxidative stress [114]. Additionally, it can interfere with antioxidant mechanisms in the central nervous system (CNS) [113]. Both commensal and pathogenic microorganisms can influence ROS levels by modulating mitochondrial activity or activating NADPH oxidases [112].

ROS enhance signaling pathways mediated by growth factors such as epidermal growth factor (EGF) and platelet-derived growth factor (PDGF). These factors promote receptor tyrosine kinase activity (EGFR and PDGFR) and their autophosphorylation, thereby activating downstream PI3K/Akt and MAPK signaling pathways that drive tumorigenesis [112].

Overexpression of EGFR, which plays a critical role in glioma development, is frequently observed in glioblastoma multiforme [115].

The MAPK pathway is modulated by the oxidation of cysteine residues, a process induced by ROS. This oxidation can activate MAP3Ks, the initial components of the pathway, through conformational changes or by enhancing kinase activity. ROS can also indirectly influence the MAPK pathway by inhibiting phosphatases [116].

The MAPK pathway has been strongly associated with glioma development [117].

Studies have shown that glioma cells accumulate large amounts of quinolinic acid (QUIN), which contributes to increased NAD+ production. This process is catalyzed by the enzyme quinolinate phosphoribosyltransferase (QPRT), whose elevated levels have been reported in highly malignant gliomas [118].

ROS can both activate and suppress the NF-κB pathway. Excessive ROS levels can lead to sustained activation of this pathway by disrupting negative feedback mechanisms. ROS influence NF-κB signaling at various points, including modifying cysteine residues in proteins such as IKK, resulting in prolonged activation [116]. Additionally, ROS activate the NF-κB pathway by promoting the phosphorylation of IκBα [119].

Gut bacteria produce metabolites involved in oxidative stress processes, including secondary bile acids. Deoxycholic acid (DCA) and lithocholic acid (LCA) are produced by *Clostridium* species [120]. DCA disrupts cell membrane integrity, leading to the production of prostaglandins and reactive oxygen species (ROS) from arachidonic acid via the activation of COX-2 and lipoxygenase. This cascade results in inflammation and DNA damage [120].

LCA also generates ROS, causing damage to the gastrointestinal epithelium, triggering inflammatory reactions, and activating the NF-κB pathway. In contrast, ursodeoxycholic acid (UDCA), produced by *Parabacteroides distasonis*, exhibits antioxidant, anti-inflammatory, and anti-apoptotic properties [120].

The gut microbiota can also counteract oxidative stress in the body, notably through the production of short-chain fatty acids (SCFAs), which possess antioxidant properties [114]. Certain SCFAs can modify mitochondrial functions and induce autophagy processes. In the case of damaged mitochondria, where excess ROS is generated, autophagy can help reduce oxidative stress within cells [121].

The CNS is particularly vulnerable to oxidative stress due to its high oxygen demand, reliance on reactive oxygen species (ROS) in signaling pathways, and high levels of redox-active transition metals and autoxidative neurotransmitters. Although CNS antioxidant mechanisms are relatively limited, they are tightly regulated. A key component of these mechanisms is superoxide dismutase (SOD), which reduces superoxide anions to molecular oxygen and hydrogen peroxide [113].

Microorganisms in probiotics can counteract elevated levels of ROS by producing antioxidative enzymes such as SOD and catalase, as well as antioxidant metabolites like folic acid and glutathione (GSH) [112]. Hydrogen, a potent antioxidant secreted by *Clostridium* species, reduces hydroxyl radicals (·OH) and contributes to oxidative stress mitigation [113].

GBM tumors exhibit altered redox homeostasis compared to physiological conditions. These cells contain hypoxic regions, contributing to tumor resistance to radio- and chemotherapy by enhancing the antioxidant system of tumor cells [122]. Addressing this issue, Sharma et al. [123] investigated the effects of kaempferol, a compound capable of modulating ROS production, on glioma cells. By stimulating intracellular oxidative stress, kaempferol induced programmed cell death in human glioma cells. This process occurred through a decrease in Bcl-2 protein expression, an increase in caspase-3 expression, and alterations in mitochondrial membrane potential. Additionally, kaempferol enhanced the cytotoxic effects of doxorubicin, further exacerbating redox imbalance [122,123].

Other anticancer compounds, including allantolactone, cannabidiol, and xanthohumol, have demonstrated similar properties. These agents induce GBM cell apoptosis through ROS production and mechanisms such as effector caspase activation [124,125,126].

## 6. Main Signaling Pathways Associated with CNS Tumors

The gut–brain axis influences tumor progression through direct microbiota interactions with the blood–brain barrier (BBB), immune modulation, and the regulation of metabolites [2,3]. Dysregulation of the BBB, associated with the zonulin factor, increases its permeability, enabling the translocation of microbiota-derived metabolites and bacterial components (e.g., lipopolysaccharides) into the brain. These molecules can activate microglia and induce chronic inflammation, thereby promoting tumorigenesis and immunosuppression (Figure 1) [3].

The gut microbiota plays a crucial role in numerous physiological and pathological processes within the host organism, influencing both health and the development of diseases, including CNS cancers [13]. By producing various metabolites, the microbiota activates multiple signaling pathways that modulate the tumor microenvironment in the brain. Key pathways include NF-κB, MAPK, PI3K/AKT/mTOR, and KP/AhR, which regulate cancer cell proliferation, angiogenesis, immunosuppression, and inflammatory responses [13,105,127].

Disruptions in microbiota composition can result in the overactivation of signaling pathways, promoting chronic inflammation that facilitates mutations and enhances cancer cell survival. Additionally, interactions between the microbiota and the immune system reduce cytotoxic lymphocyte activity and increase the population of regulatory T cells (Tregs), further promoting immunosuppression and tumor development [13,128].

Understanding the mechanisms underlying these pathways is critical for identifying novel therapeutic targets and exploring opportunities to modulate the microbiota in the treatment of CNS cancers.

### 6.1. NF-κB Pathway

The activation of the nuclear factor kappa B (NF-κB) pathway plays a pivotal role in carcinogenesis by promoting the mesenchymal phenotype of glioblastoma multiforme, which is characterized by aggressive tumor progression [129]. Numerous studies have demonstrated that activation of this pathway is a common feature in glioblastoma multiforme [130,131,132,133,134,135,136,137,138].

Bacterial-derived ligands, such as peptidoglycans (e.g., muramyl dipeptide, MDP), activate microglia through the nucleotide-binding oligomerization domain 2 (Nod2) receptor, while lipopolysaccharides bind to toll-like receptor 4 (TLR4). These interactions lead to the activation of NF-κB and MAPK pathways, resulting in the secretion of pro-inflammatory cytokines, including TNF-α, IL-1β, IL-6, and IL-8. This pro-inflammatory response fosters a tumor-promoting microenvironment in the CNS [13,139].

Under the influence of specific stimulatory factors, certain kinases are activated, leading to the phosphorylation of the NF-kappa-B inhibitor alpha (IκBα) associated with NF-κB. Phosphorylated IκBα undergoes ubiquitination and degradation in the proteasome, freeing NF-κB. The released NF-κB translocates to the nucleus, where it activates the transcription of inflammatory genes such as IL-6 and TNF-α [140].

For muramyl dipeptide (MDP), a clathrin- and dynamin-dependent endocytic pathway facilitates its internalization and subsequent Nod2 activation [141]. Upon recognizing MDP, the Nod2 receptor triggers NF-κB activation by engaging the serine-threonine kinase (RICK), which subsequently recruits transforming growth factor β-activated kinase 1 (YES1) [141,142,143].

**Figure 2:**
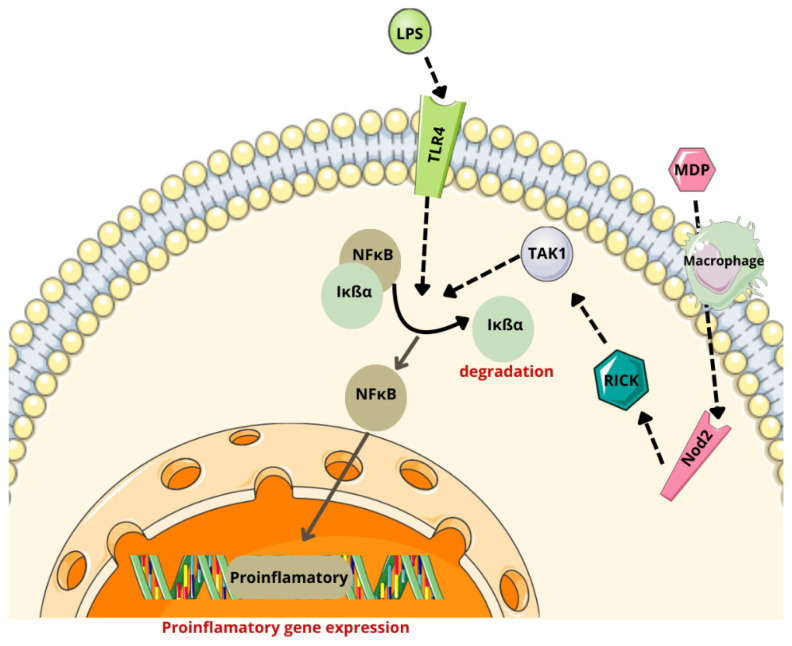
NF-κB pathway activated by bacterial ligands. (MDP—muramyl dipeptide; LPSs—lipopolysaccharides; TLR4—toll-like receptor 4; Nod2—nucleotide-binding oligomerization domain 2 receptor; RICK—serine-threonine kinase; TAK1—transforming growth factor β-activated kinase 1; NF-κB—nuclear factor kappa B; IκBα—NF-kappa-B inhibitor alpha). Parts of the figure were drawn by using pictures from Servier Medical Art, licensed under Creative Commons Attribution 4.0 International.

Chronic NF-κB activation induces persistent inflammation that promotes tumor cell proliferation and survival, contributing to tumor progression (Figure 2) [13,144].

### 6.2. MAPK Pathway

The mitogen-activated protein kinase (MAPK) signaling pathway is critical for promoting inflammatory processes in the tumor microenvironment and facilitating immune evasion [145]. It achieves this through the paracrine and autocrine release of tumor proliferation factors and cytokines, as well as by sustaining cellular proliferation and reducing apoptosis in tumor cells [145]. This pathway has been implicated in glioblastoma development [117].

The MAPK pathway consists of a three-tier kinase cascade involving MAP3K, MAP2K, and MAPK. MAP3Ks, such as TAK1 and RAF [146,147], phosphorylate and activate MAP2K, which in turn phosphorylates and activates MAPK [148,149,150]. Microbial metabolites, including LPSs and MDPs, can activate this pathway by engaging surface receptors such as TLR4 and Nod2 [151,152].

These receptors initiate the pathway by activating MAP3K. Macrophages internalize MDPs into the cytosol [141,151,152], where they are recognized by Nod2. Nod2 then activates MAP2K through the recruitment of RICK and TAK1 [142]. Once MAP2K is activated, it phosphorylates and activates MAPK [149].

**Figure 3:**
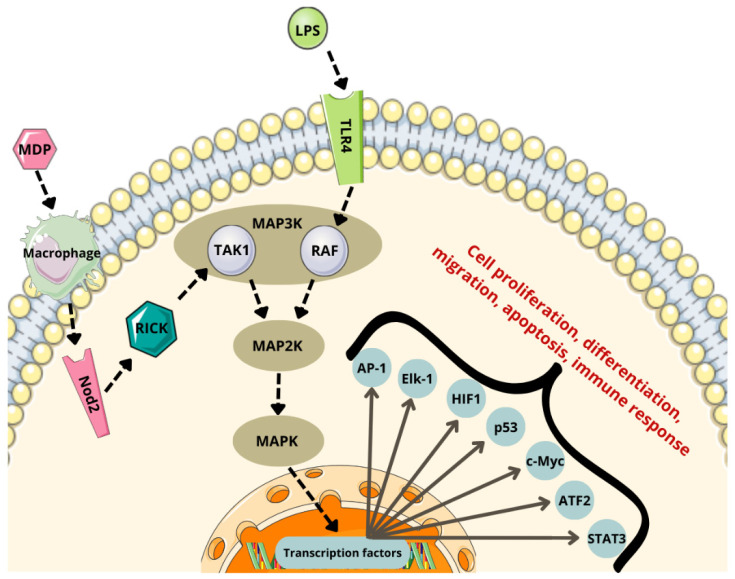
MAPK pathway activated by bacterial ligands. (MDP—muramyl dipeptide; LPSs—lipopolysaccharides; TLR4—toll-like receptor 4; Nod2—nucleotide-binding oligomerization domain 2 receptor; RICK—serine-threonine kinase; TAK1—transforming growth factor β-activated kinase 1; RAF—rapidly accelerated fibrosarcoma; MAPK—mitogen-activated protein kinase; MAP2K—mitogen-activated protein kinase 2; MAP3K—mitogen-activated protein kinase 3; AP-1—activator protein-1; Elk-1—e-twenty-six (ETS)-like transcription factor 1; HIF1—hypoxia-inducible factor 1; p53—tumor protein P53; c-Myc—cellular myelocytomatosis oncogene; ATF2—activating transcription factor 2; STAT3—signal transducer and activator of transcription 3). Parts of the figure were drawn by using pictures from Servier Medical Art, licensed under Creative Commons Attribution 4.0 International.

The MAPK pathway activates various substrate proteins, including transcription factors such as activator protein-1 (AP-1), E-twenty-six (ETS)-like transcription factor 1 (Elk-1), hypoxia-inducible factor 1 (HIF1), activating transcription factor 2 (ATF2), tumor protein p53 (p53), cellular myelocytomatosis oncogene (c-Myc), and signal transducer and activator of transcription 3 (STAT3). These transcription factors regulate the expression of genes associated with cancer cell proliferation and survival (Figure 3) [149,153].

AP-1 is a transcription factor complex composed of Fos and Jun proteins that activate genes associated with cell proliferation and survival [153]. AP-1 promotes the transcription of cell cycle-related genes such as cyclin D1 [153].

Elk-1, phosphorylated by ERK, regulates genes involved in cell differentiation and growth, such as c-Fos [154]. Hypoxia-inducible factor 1 (HIF1) controls the expression of genes related to glucose metabolism (e.g., GLUT1, HK2) and angiogenesis (e.g., VEGF), thereby supporting survival and growth of gliomas under hypoxic conditions. Additionally, ERK stabilizes c-Myc, enhancing the transcription of genes that drive cell proliferation, including those associated with nucleotide synthesis and glucose metabolism [155].

Signal transducer and activator of transcription 3 (STAT3) promotes angiogenesis, and elevated STAT3 expression levels in gliomas correlate with unfavorable prognostic outcomes [156]. Phosphorylated STAT3 (p-STAT3) in human gliomas influences inflammatory responses, with its expression varying significantly across glioma types and stages of pathology. This variability correlates with the degree of immune cell infiltration [156].

The STAT3 signaling pathway plays a crucial role in regulating EGFR-associated adhesion molecules and monocyte adhesion in GBM. Specifically, vascular cell adhesion molecule-1 (VCAM-1) expression is mediated by the p38/STAT3 signaling pathway. Hyperactivation of STAT3 has been shown to enhance tumor invasiveness by promoting the secretion of matrix metalloproteinases and upregulating focal adhesion kinase (FAK). The FAK/STAT3 signaling pathway is implicated in the migratory capacity of GBM cells and the production of interleukin-8 (IL-8) [156,157,158,159].

The p53 protein exerts a suppressive effect on tumorigenesis by modulating programmed cell death, autophagy, the cell cycle, and DNA repair mechanisms [160]. In most cellular phenotypes, p53 inhibits glycolytic pathways while promoting oxidative phosphorylation (OXPHOS). It achieves this by upregulating cytochrome c oxidase subunits 1 (Cox1p) and 2 (Cox2p), which are essential for the transfer of electrons from complex III to complex IV in the electron transport chain (ETC) [161,162].

Complex IV, also known as cytochrome c oxidoreductase, serves as the terminal enzyme of the ETC. It is responsible for the enzymatic reduction of diatomic oxygen, a critical step in cellular respiration, facilitated by its prosthetic groups and essential cofactors [161,162].

Moreover, the p53 protein inhibits the transcriptional activity of NF-κB by suppressing IκBα and IκB kinases [163]. The status of p53 has also been shown to correlate with disease progression and survival rates in patients with glioblastoma multiforme undergoing radiotherapy and chemotherapy [164].

### 6.3. PI3K/Akt/mTOR Pathway

The phosphoinositide 3-kinase (PI3K)/protein kinase B (Akt)/mammalian target of rapamycin (mTOR) signaling cascade plays a pivotal role in regulating the proliferation, survival, and metabolic processes of cancer cells, particularly gliomas [150]. The gut microbiota, through its metabolic byproducts and interactions with the immune system, influences the activation of this signaling pathway, thereby affecting the etiology and progression of brain tumors [150,165].

**Figure 4:**
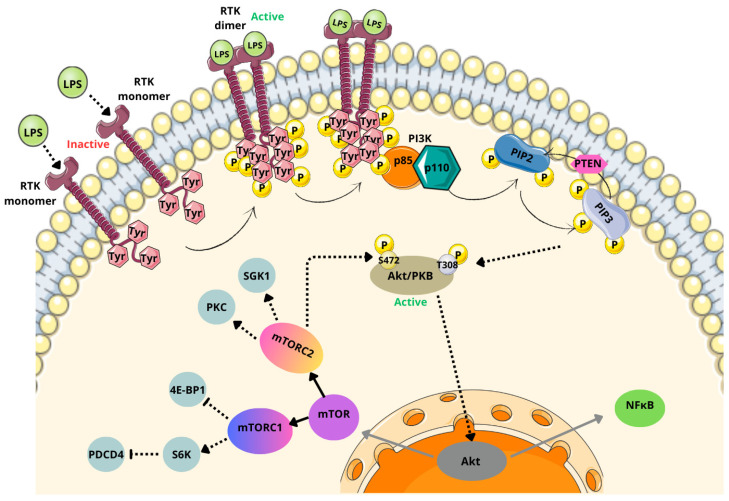
PI3K/Akt/mTOR pathway activated by bacterial ligands. (LPSs—lipopolysaccharides; RTK—receptor tyrosine kinase; Tyr—tyrosine; P—phosphor; PI3K—phosphoinositide 3-kinase; p85—regulatory subunit p85 of PI3K; p110—catalytic subunit of PI3K; PIP2—phosphatidylinositol-4, 5-bisphosphate; PIP3—phosphatidylinositol-3,4, 5-triphosphate; PTEN—phosphate and tensin homolog deleted on chromosome 10; Akt/PKB—protein kinase B; T308—threonine at position 308; S472—serine at position 473; mTOR—mammalian target of rapamycin; mTORC1—mammalian target of rapamycin complex 1; mTORC2—mammalian target of rapamycin complex 2; S6K—ribosomal protein kinase; PDCD4—programmed cell death protein 4; 4E-BP1—eukaryotic translation initiation factor 4E protein-binding 1; PKC—protein kinase C; SGK1—serum and glucocorticoid-induced protein kinase 1; NFκB—nuclear factor kappa B). Parts of the figure were drawn by using pictures from Servier Medical Art, licensed under Creative Commons Attribution 4.0 International.

The PI3K/Akt/mTOR signaling cascade is initiated when ligands, such as LPSs, interact with receptor tyrosine kinases (RTKs) (Figure 4) [150].

This interaction leads to the activation of RTKs, where RTK monomers dimerize, and tyrosine residues in their intracellular domains undergo phosphorylation. This, in turn, triggers the enzyme phosphoinositide 3-kinase (PI3K). Activated PI3K catalyzes the transformation of phospholipids in the cell membrane, particularly in class I PI3Ks (the most critical class for tumorigenesis) [150,166].

Class I PI3Ks consist of a catalytic subunit (p110) and a regulatory subunit (p85) [167]. These enzymes mediate the conversion of phosphatidylinositol-4,5-bisphosphate (PIP2) into phosphatidylinositol-3,4,5-triphosphate (PIP3). During this process, the p85 subunits of PI3K dimerize and release their p110 subunit, allowing the membrane protein to phosphorylate PIP2 into PIP3. The phosphate and tensin homolog deleted on chromosome 10 (PTEN) acts antagonistically to PI3K by reducing PIP3 levels and inhibiting Akt/PKB activity [150,167].

In the next step of the signaling cascade, PIP3 activates protein kinase B (Akt/PKB) by phosphorylating threonine at position 308 (T308) and serine at position 473 (S473). The phosphorylation of S473 also requires mTORC2, after which Akt/PKB becomes fully activated [115,167].

In its inactive form, Akt/PKB exists in the cytosol as a complex with heat shock protein 27 (Hsp27). Upon activation, Hsp27 is phosphorylated and dissociates from the complex, allowing Akt to translocate to the nucleus. In the nucleus, Akt modulates the expression of various proteins, including mTOR and NF-κB [150,167].

Phosphorylation of the mTOR signaling pathway is a critical factor in microglial activation. Furthermore, the mTOR pathway plays an integral role in modulating NF-κB activity and the inflammatory response [144].

mTOR serves as a metabolic switch between catabolism and anabolism, functioning within two distinct complexes: mTORC1 (complexed with the Raptor protein) and mTORC2 (complexed with the Rictor protein) [168].

mTORC1 regulates metabolic processes and cell proliferation via downstream effectors such as S6 ribosomal protein kinase (S6K) and eukaryotic translation initiation factor 4E binding protein 1 (4E-BP1). These effectors accelerate glial cell growth, facilitate efficient progression through the G1 phase of the cell cycle, and modulate programmed cell death protein 4 (PDCD4) [168,169]. Additionally, mTORC1 inhibits autophagy, enabling gliomas to store cellular resources and avoid degradation [170].

Once activated, mTORC1 phosphorylates various downstream substrates, with its primary function being the activation of S6K1. The activated form of S6K1 suppresses programmed cell death protein 4 (PDCD4) [168].

mTORC2 plays a fundamental role in regulating cytoskeletal dynamics, cell survival, proliferation, metabolism, and angiogenesis [169]. It achieves this through the phosphorylation of targets such as protein kinase B (Akt), serum- and glucocorticoid-induced protein kinase 1 (SGK1), and protein kinase C (PKC). Upon activation, mTORC2 phosphorylates Akt at Ser473, a critical step for full Akt activation. Additionally, mTORC2 interacts with and modulates PKC activity, which is essential for cytoskeletal reorganization [171,172].

Another important target of mTORC2 is SGK1, a protein integral to ion transport processes [168,171,173]. The role of SGK1 in promoting the growth and survival of GBM stem cell lines has been confirmed in studies involving multiple patient-derived cell lines [171].

### 6.4. Kynurenine/Ahr Pathway

**Figure 5:**
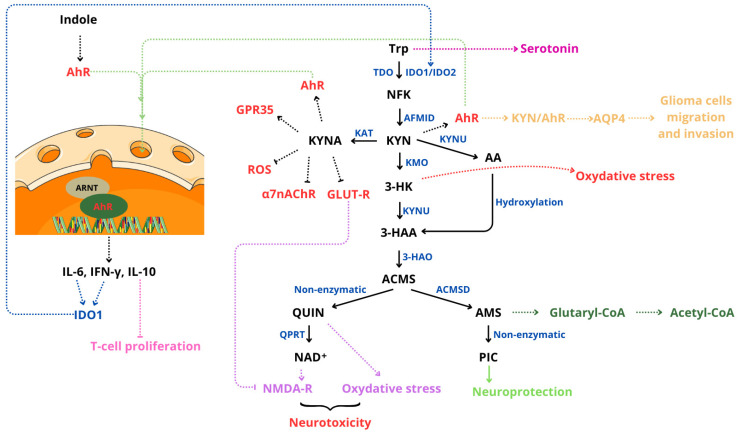
Kynurenine/AhR pathway. (Trp—tryptophan; TDO—tryptophan 2,3-dioxygenase; AA—anthranilic acid; 3-HAA—3-hydroxyanthranilic acid; IDO1/2—indoleamine 2,3-dioxygenase 1/2; NFK—n-formylkynurenine; AFMID—kynurenine formamidase; KYN—kynurenine; KAT—kynurenine transaminase; KYNA—kynurenic acid; KYNU—kynureninase; AA—anthranilic acid; KMO—kynurenine monooxygenase; 3-HK—3-hydroxykynurenine; 3-HAA—3-hydroxyanthranilic acid; 3-HAO—3-hydroxyanetanilic acid 3,4-dioxanase; ACMS—2-amino-3-carboxyconic acid 6-semialdehyde; QUIN—quinolinic acid; QPRT—quinoline phosphoribosyltransferase; NAD+—nicotinamide adenine dinucleotide; ACMSD—α-amino-β-carboxycoate semialdehyde decarboxylase; AMS—2-aminomuconate-6-semialdehyde; PIC—picolinic acid; AhR—aryl hydrocarbon receptor; ARNT—nuclear translocator protein AhR; GPR35—G35 protein-coupled receptor; ROS—reactive oxygen species; α7nAChR—receptor α7-nicotinic acetylcholine; GLUT-R—ionotropic glutamate receptors; NMDA-R—N-methyl-D-aspartate receptor; AQPR—aquaporin-4; Glutaryl-CoA—glutaryl-coenzyme A; Acetyl-CoA—acetyl coenzyme A; IL-6—interleukin-6; IFN-γ—interferon gamma; IL-10—interleukin-10). Parts of the figure were drawn by using pictures from Servier Medical Art, licensed under Creative Commons Attribution 4.0 International.

The kynurenine pathway (KP) is the primary metabolic route of tryptophan in the human body, playing a significant role in regulating immune responses, neuronal functionality, and tumor development, particularly in CNS tumors (Figure 5) [14].

Tryptophan is metabolized into various neuroactive compounds, including kynurenine (KYN), quinolinic acid (QUIN), and kynurenic acid (KYNA). These metabolites have both neuroprotective and neurotoxic properties [14,173].

KP activity is upregulated in response to pro-inflammatory cytokines, such as interferon-gamma (IFN-γ) and tumor necrosis factor (TNF), through the induction of indoleamine 2,3-dioxygenase 1 (IDO-1) [14,173]. The intestinal microbiota also has an impact on tryptophan metabolism via modulating its availability for the kynurenine pathway. Certain bacterial species metabolize tryptophan into indoles and their derivatives, which then interact with the aryl hydrocarbon receptors (AhRs) in the CNS [14,173].

Overexpression of key KP enzymes, including IDO and tryptophan 2,3-dioxygenase (TDO), is frequently observed in CNS tumor cells, such as gliomas, leading to significantly elevated levels of kynurenine [14,173].

Ligands for AhR, such as kynurenine, indole-3-carbinol, and various indole derivatives, activate this receptor by binding to it. Once activated, AhR translocates to the nucleus and forms a complex with the AhR nuclear translocator protein (ARNT). This complex functions as a transcription factor, triggering the expression of genes involved in immunosuppression, angiogenesis, and tumor proliferation [173].

The AhR-ARNT complex regulates the expression of interleukin-6 (IL-6) in macrophages, interleukin-10 (IL-10) in natural killer (NK) cells and dendritic cells, and interferon-gamma (IFN-γ). Additionally, IL-6 subsequently induces the activation of indoleamine 2,3-dioxygenase-1 (IDO-1) [173].

Kynurenine inhibits the activity of T cells, particularly CD4+ and CD8+ cells, while promoting the differentiation of regulatory T cells (Tregs) through AhR activation. This mechanism facilitates tumor evasion from the immune system and impairs the functionality of natural killer (NK) cells. In environments with elevated kynurenine levels, T cells and NK cells show increased susceptibility to apoptosis, a process linked to oxidative stress induced by kynurenine metabolites, such as 3-hydroxykynurenine (3-HK) [173,174].

Dysbiosis of the gut microbiota has been associated with inflammatory processes and alterations in tryptophan metabolism, which can disrupt BBB integrity. These disruptions increase the permeability of pro-inflammatory cytokines and kynurenine metabolites, collectively fostering a tumor-promoting microenvironment by suppressing antitumor immunity [14,15].

The kynurenine pathway begins with the oxidation of tryptophan (Trp) to N-formylkynurenine (NFK), catalyzed by the enzymes indoleamine-2,3-dioxygenase (IDO1/IDO2) or tryptophan-2,3-dioxygenase (TDO). In the next step, NFK is converted to kynurenine (KYN) by kynurenine formamidase (AFMID). Kynurenine is subsequently metabolized through two distinct pathways, one of which involves its enzymatic conversion to kynurenic acid (KYNA) by kynurenine transaminase (KAT). KYNA possesses neuroprotective properties, but excessive levels can inhibit NMDA receptors, potentially leading to disruptions in neuronal signaling [15,175].

Kynurenine can also undergo transformation into 3-hydroxykynurenine (3-HK) through the action of kynurenine monooxygenase (KMO). 3-HK is recognized for its neurotoxic properties and its ability to induce oxidative stress by generating free radicals, such as superoxide (O_2_^−^) and hydrogen peroxide (H_2_O_2_) [176].

Additionally, kynurenine can be metabolized by kynureninase (KYNU) to produce anthranilic acid (AA), which can be further hydroxylated to form 3-hydroxyanthranilic acid (3-HAA), a metabolite with potential implications for both neurotoxicity and immune regulation [15].

This compound is subsequently oxidized by 3-hydroxyanthranilic acid 3,4-dioxygenase (3-HAO) to produce the unstable intermediate 2-amino-3-carboxymuconic acid 6-semialdehyde (ACMS), which can cyclize to form quinolinic acid (QUIN). QUIN is further metabolized by quinolinate phosphoribosyltransferase (QPRT) to generate nicotinamide adenine dinucleotide (NAD+) [15,173,174,177].

Additionally, ACMS can be converted by α-amino-β-carboxymuconate semialdehyde decarboxylase (ACMSD) into 2-aminomuconate-6-semialdehyde (AMS). AMS can then either cyclize to form picolinic acid (PIC) or be metabolized by AMS-dehydrogenase (AMSD) to produce acetyl-coenzyme A (acetyl-CoA) [15,173,174].

QUIN, as an activator of NMDA receptors, induces toxic levels of calcium ions within cells, leading to mitochondrial production of free radicals, DNA damage, and the activation of poly(ADP-ribose) polymerases (PARP). The resulting production of ROS facilitates lipid peroxidation, further exacerbating cellular damage [15]. Additionally, QUIN inhibits glutamate uptake into synaptic vesicles and suppresses astroglial glutamine synthetase activity, resulting in elevated extracellular glutamate levels. This glutamate accumulation contributes to neurotoxicity by overstimulating excitatory signaling pathways [15].

Kynurenines exhibit the ability to both generate and eliminate reactive oxygen and nitrogen species. KYN displays dual properties: it facilitates the formation of reactive species such as H_2_O_2_, OH∙, and ONOO^−^ while simultaneously scavenging O_2_^−^ and H_2_O_2_ [176].

Kynurenic acid acts as an antagonist of both quinolinic acid (QUIN) and picolinic acid (PIC), mitigating their neurotoxic effects [177]. Additionally, KYNA serves as an inhibitor of the α7 nicotinic acetylcholine receptor (α7nAChR), a key player in the central cholinergic anti-inflammatory pathway [176]. Furthermore, KYNA antagonizes glutamate receptors (GLUT-R) by inhibiting three ionotropic receptors, N-methyl-D-aspartate (NMDA) receptors, kainic acid receptors (KAR), and AMPA receptors, thereby exerting neuroprotective effects [165].

KYNA has been shown to act as an agonist of the G-protein-coupled receptor 35 (GPR35), reducing calcium mobilization through this receptor. This reduction in calcium mobilization decreases mitochondrial damage, leading to lower reactive oxygen species (ROS) production and inhibition of NLRP3 inflammasome activation [178].

QUIN undergoes catabolism, while PIC is synthesized in human neuronal cells. Interestingly, neuroblastoma cell lines display a preference for QUIN production and PIC utilization. Dysregulation of the kynurenine pathway, particularly reduced expression of ACMSD, may selectively shift tryptophan metabolism toward QUIN generation. This shift increases intracellular NAD^+^ levels at the expense of neuroprotective and potentially antitumor PIC. Such metabolic reprogramming may contribute to tumor survival by enhancing DNA repair efficiency through increased availability of NAD^+^ for PARP activation [175]. Moreover, Du et al. [179] identified the KYN-AhR-AQP4 signaling cascade, which exacerbates glioma malignancy by regulating aquaporin-4 (AQP4)-mediated migration and invasion of glioma cells [167].

## 7. Therapeutic Perspectives

The wide variety of CNS cancers, coupled with their high mortality rates and the challenges in treatment, underscores the need for developing more effective and precise therapeutic approaches [180]. Immune system-targeted therapies and treatments with signaling pathway inhibitors show considerable potential.

As discussed earlier in this review, the microbiota significantly influences tumorigenesis, either by promoting or inhibiting tumor development. Notably, the gut bacterial composition of GBM patients differs from that of healthy individuals, with an observed increase in bacterial diversity and alterations in the abundance of specific bacterial taxa [180].

Modulation of the gut microbiota is increasingly being explored as a strategy to enhance the treatment of gliomas and other conditions. Interventions such as fecal microbiota transplantation (FMT), probiotics, prebiotics, symbiotic, postbiotics, and dietary modifications are being investigated to support a healthy gut microbiome composition [181,182]. A recent publication highlights ongoing clinical trials examining the role of gut microbiome modulation in cancer therapies [183].

Probiotics can enhance standard therapies by boosting the immune response and modifying the tumor microenvironment, particularly when used alongside immune checkpoint inhibitor (ICI) therapy. Notably, probiotics such as *Bifidobacterium lactis* and *Lactobacillus plantarum* have been shown to reduce tumor volume and extend survival in glioma mouse models. This effect is thought to occur through the suppression of the PI3K/AKT signaling pathway, which is crucial for tumor growth, and by modulating the gut microbiota. These probiotics help increase the number of beneficial bacteria like *Lactobacillus* while decreasing potential pathogens. While these mouse model results are promising, additional clinical trials are necessary to confirm their efficacy in humans [184].

The probiotic *Bifidobacterium* has been shown to reduce glioma growth by inhibiting the MEK/ERK signaling pathway. Additionally, it enhances the diversity of the tumor microbiota and increases the abundance of *Bifidobacterium* in the intestine, presenting new perspectives in glioma therapy [185].

The gut–brain axis plays a significant role in diffuse brainstem glioma, particularly in children. Some gut bacteria have been associated with increased disease progression, while others may slow it down. Administering probiotics could help restore the normal Firmicutes/Bacteroidetes ratio, which has been linked to glioma progression [186]. Studies indicate that glioma growth influences the populations of *Bacteroidia* and *Firmicutes* bacteria in the gut microbiome of mice. Furthermore, the abundance of *Bacteroidia* can directly impact glioma growth. Dysbiosis, in particular, can downregulate the expression of forkhead box P (Foxp3) in the brain, contributing to glioma development. In contrast, fecal microbiota transplantation (FMT) has been shown to replenish the microbiota and slow glioma growth [187].

FMT is a procedure that involves transferring intestinal microflora from a healthy donor to a patient in order to modulate the patient’s microbiota [188]. FMT is currently being explored as a therapeutic approach to enhance the composition of the gut microbiota in individuals diagnosed with glioblastoma. By transplanting healthy or optimized gut microflora, FMT aims to improve the immune response in these patients, potentially increasing T-cell activation. Preliminary results suggest that combining FMT with ICI therapy in selected glioma patients enhances treatment efficacy [189].

Postbiotics are emerging as a valuable support in immune checkpoint inhibitor therapies. One example is the ROSALIE clinical trial, which is evaluating EO2401, an innovative peptide vaccine derived from the gut microbiota. This vaccine is being tested both as a monotherapy and in combination with nivolumab or nivolumab and bevacizumab in patients with progressive glioblastoma multiforme [190].

Glioma has been shown to affect fecal metabolite levels [191]. In both mice and humans with glioma, levels of noradrenaline and 5-hydroxyindoleacetic acid (5-HIAA) decrease as tumor growth progresses. However, treatment with temozolomide has been observed to mitigate these changes [191].

Additionally, a study in glioma-bearing mice treated with antibiotics (ABX) demonstrated that antibiotic therapy altered the gut microbiota composition, reduced the number of NK cell subsets, and affected the expression of inflammatory and homeostatic proteins in microglia. These changes were associated with intracranial glioma growth, highlighting the critical role of gut microbiota composition in supporting an effective anti-tumor immune response [192].

Substances produced by the microbiota can significantly modulate the body’s response to cancer [112,114]. An increase in SCFA levels, such as acetate and propionate, enhances the efficiency of cellular antioxidant mechanisms. Therefore, it can be hypothesized that increasing the population of SCFA-producing intestinal bacteria may support cancer therapy [112,114]. This can be achieved by providing key microbial substrates, such as carbohydrates and glycans, through the diet in the form of prebiotics. Such modifications to the microbiome composition can reduce inflammation and lower ROS levels in the intestines, contributing to a more favorable environment for therapeutic outcomes [112,114]. SCFAs also play a role in immune cell differentiation, influencing T and B lymphocytes and thereby regulating immune responses [188].

Certain bacterial species have shown potential in anticancer strategies through their production of unique virulence factors [193]. *Pseudomonas aeruginosa* releases a potent virulence factor capable of destroying cancer cells via ADP-ribosylation of the eukaryotic elongation factor 2 (eEF2). Additionally, Pseudomonas exotoxin can be conjugated with antibodies to enhance precision in targeting cancer cells [193].

*Streptococcus pyogenes* produces arginine deiminase, an enzyme that depletes arginine, an amino acid critical for cancer cell survival [193]. Similarly, *Escherichia coli* generates cytotoxic necrosis factor 1 (CNF1), which has demonstrated antitumor activity in mouse glioma models by reducing tumor size while preserving neuronal cell viability during the symptomatic phase [194].

These findings highlight the therapeutic potential of leveraging bacterial metabolites and toxins for targeted cancer treatment.

CNF1, when administered intracerebrally, significantly prolonged the survival of mice with glioma. Remarkably, it demonstrated rapid and long-lasting effects after a single dose, highlighting its potential importance as an effective treatment option [195].

The microbiota plays a significant role in immunomodulation, as bacterial peptides have been shown to be recognized by tumor-infiltrating lymphocytes (TILs), stimulating their activity. This interaction enhances the immune response to glioma, contributing to improved anti-tumor efficacy [188,196].

GBM is characterized by innate or acquired resistance to chemotherapy, prompting the exploration of various treatment modifications. GBM demonstrates NF-κB activity, which is associated with therapeutic resistance [138]. In a study comparing the NF-κB pathway inhibitor 11-7082 with temozolomide, the inhibitor was shown to suppress pathway activity, enhance apoptosis, and reduce tumor viability [138]. Additionally, radiotherapy combined with temozolomide has demonstrated a beneficial effect on survival in patients with newly diagnosed glioma [197].

The use of probiotics has shown potential in improving the efficacy of radiation therapy, which is often accompanied by negative side effects such as disruption of the gut microbiota. Studies suggest that certain gut bacteria, particularly *Firmicutes* and *Bacteroides*, may positively influence the outcome of radiotherapy [198]. Microbial metabolites, such as butyric acid derivatives, have been shown to inhibit proliferation and induce apoptosis in glioma cell lines, thereby enhancing the effects of radiation therapy [199,200]. Additionally, sodium butyrate can inhibit glioma cell growth and invasiveness by affecting the cell cycle [201]. However, there are also studies exploring the pro-carcinogenic effects of butyrate, as gut bacteria in colorectal cancer patients may contribute to tumorigenesis and cellular aging through the production of butyrate [202].

Probiotics can also support chemotherapy by minimizing side effects and improving treatment efficacy. In a mouse model of pancreatic ductal adenocarcinoma, the combination of chemotherapeutic agents with *Lactobacillus* probiotics was shown to inhibit disease progression and have a favorable effect on liver enzyme levels [203].

Another promising approach in glioma therapy is the use of ICIs [189]. The gut microbiota plays a critical role in this context, as it can reduce immune suppression and enhance immune cell and antibody production in glioma, thereby improving treatment outcomes. The appropriate composition of the microbiota modulates the immune system’s response to inflammation by stimulating immune cell activity and promoting a more effective anti-tumor response [189].

The microbiota can influence the efficacy of chemotherapeutic treatments, enhancing the effects of the drugs administered [204,205]. For example, in lung adenocarcinoma in mice, it was demonstrated that the bacteria *Diaphorobacter nitroreducens*, when combined with oxaliplatin, increased the number of CD163- and CD68-positive macrophages while reducing Treg cells (CD4 FOXP3), thus slowing tumor progression [204]. Additionally, disruption of the microbiota due to antibiotic use has been associated with the advancement of glioma progression and reduced effectiveness of temozolomide chemotherapy [205].

Ursodeoxycholic acid (UDCA) has also been shown to exhibit anti-tumor activity, particularly against GBM cells [107]. Studies have demonstrated that UDCA reduces the viability of GBM cells and increases the expression of genes associated with apoptosis and cell cycle arrest. It also induces mitochondrial damage and enhances ROS production in GBM cells. Furthermore, UDCA initiates endoplasmic reticulum (ER) stress; however, this stress alone is insufficient to overcome glioma cells. The effect was significantly enhanced when bortezomib was used in combination with UDCA, as bortezomib prolonged ER stress, leading to the inhibition of GBM progression [107].

Inhibition of EGFR, which activates downstream signaling pathways such as PI3K/Akt/mTOR, has been considered a promising therapeutic target. However, clinical trials have not produced the anticipated results [115]. Various EGFR inhibitors, including gefitinib, erlotinib, temozolomide, and irinotecan, have been tested. The lack of success in these therapies is attributed to the persistence of pathway activity despite EGFR dephosphorylation, indicating that other elements of the signaling cascade remain active. These findings underscore the need for further research to identify more effective strategies to target this pathway [115].

A study investigating changes in the intestinal microbiota of mice undergoing oncolytic virus treatment for GBM demonstrated that the gut microbiota may influence therapy outcomes. Higher levels of *Bifidobacterium* were associated with an enhanced immune response, leading to greater efficacy of viral therapy [206].

Bacterial drug transporters present an intriguing therapeutic approach, especially for overcoming the challenges posed by the blood–brain barrier in delivering drugs to gliomas [207]. A novel system using “Trojan bacteria”, constructed with bacteria loaded with glucose polymers and photosensitive silicon nanoparticles (ICG), has been developed. These nanoparticles can be internalized by attenuated *Salmonella typhimurium* VNP20009 (VNP) or *Escherichia coli* 25922 (EC). This carrier system is activated by 808 nm laser radiation, causing targeted damage to cancer cells while inducing the rupture of bacterial cells. The lysates of both cancer and bacterial cells produced after photothermal treatment have been shown to stimulate an immune response, offering a potential enhancement to anticancer therapy [207].

In the context of future anticancer therapies, the potential of *Lactiplantibacillus plantarum* T1 deserves attention. The cell-free supernatant of this strain has demonstrated anti-inflammatory and antioxidant properties by inhibiting the MAPK and NF-κB pathways in LPS-stimulated RAW264.7 mouse macrophages [208].

In a study involving mice with LPS-induced endotoxin shock, lipoteichoic acid produced by *Lactiplantibacillus plantarum* significantly reduced excessive TNFα production, thereby improving survival rates. This response involved the inhibition of MAPK and NF-κB signaling pathways, which are also critical drivers of tumorigenesis [209]. These findings suggest that *Lactiplantibacillus plantarum* could play a role in modulating key pathways involved in both inflammation and cancer progression [208,209].

It is also worth noting other mechanisms that may be relevant for anticancer therapy, particularly those involving the gut microbiota [65,210,211,212]. GBMs often adapt to chemo- or radiotherapy by interfering with tryptophan metabolism [210]. Tumors exploit tryptophan catabolism, mediated by IDO1 and TDO, to produce kynurenine, an endogenous ligand of the aryl hydrocarbon receptor (AhR). This process suppresses the immune response, facilitating tumor progression. Inhibition of the kynurenine pathway using TDO inhibitors has shown promise in inducing immune-mediated tumor rejection, suggesting a potentially safe and effective approach to cancer immunotherapy [211,212,213]. Additionally, IDO1/TDO expression has been linked to tumor malignancy, with patients exhibiting positive IDO1/TDO protein expression having shorter survival times. IDO1 and TDO contribute to increased kynurenine production and AhR expression, which further regulate glioma cell migration and invasion. These findings highlight the potential of targeting the KP in GBM as a novel therapeutic strategy [179].

Glioma cells depend on exogenous cysteine and, under conditions of cysteine deficiency, are unable to convert methionine into cysteine. Studies have shown that cysteine deprivation reduces glioma cell viability, while a cysteine-free diet increases cell susceptibility to oxidative stress, leading to improved outcomes in a mouse model of glioma [214]. Additionally, methionine plays a crucial role in cancer cell proliferation; deficiency in both methionine and cysteine inhibits proliferation and elevates ROS levels [215]. A high-fat diet has been linked to a more aggressive form of glioma by promoting the progression and renewal of glioma cells [216]. Conversely, shifting to a ketogenic diet (KD) is considered a potential therapeutic strategy. Studies have shown that mice on a KD not only survived longer compared to controls but also experienced reduced ROS levels and enhanced outcomes from radiotherapy [217]. While the efficacy of this dietary approach is promising, researchers are exploring less demanding alternatives such as the high-fat, low-carbohydrate diet (sFHLC), which has also demonstrated potential in negatively impacting gliomas and boosting patient survival [218].

## 8. Conclusions

The interplay between the gut microbiota, oxidative stress, and CNS cancers underscores the complexity of tumor biology, positioning the microbiota as a critical regulator of CNS homeostasis and disease progression.

The microbiota–gut–brain axis plays a pivotal role in shaping immune responses, modulating signaling pathways, and maintaining oxidative balance, thereby contributing to the development of gliomas and other CNS malignancies. Dysregulation of key signaling pathways such as NF-κB, MAPK, PI3K/Akt/mTOR, and Kynurenine/AhR highlights how microbiota-driven mechanisms promote tumor growth and facilitate immune evasion.

Therapeutic approaches targeting the gut microbiota, including microbiota-modulating strategies and their integration with advanced immunotherapies, present a promising frontier in CNS cancer treatment. By addressing microbiota dysbiosis and oxidative imbalance, these strategies have the potential to enhance therapeutic efficacy, overcome resistance, and improve patient outcomes.

Further research is essential to fully explore and harness the therapeutic potential of the gut microbiota in combating CNS cancers.

## Figures and Tables

**Table 1:** Bacterial composition of intestinal bacteria and their functions.

Phylum	% Microbiome	Genus	Relevant Function	References
*Firmicutes*	60–65%	*Bacillus* *Clostridium* *Dialister* *Enterococcus* *Faecalibacterium* *Lactobacillus* *Roseburia* *Ruminicoccus* *Staphylococcus*	Some are involved in the production of short-chain fatty acids (SCFAs)	[24,27,28,29,30,31,33,34]
*Bacteroidetes*	20–25%	*Alistipes* *Bacteroides* *Parabacteroides* *Prevotella* *Sphingobacterium* *Tannerella*	Some are involved in the production of SCFAs	[24,27,28,29,30,31,33,34]
*Proteobacteria*	5–10%	*Bilophila* *Desulfovibrio* *Escherichia* *Helicobacter* *Shigella*	Signal microbial dysbiosis—for a healthy person this makes up a small part of the intestinal microbiota	[24,25,26,27,28,29,30,31,33,35]
*Actinobacteria*	3%	*Atopobium* *Bifidobacterium* *Corynebacterium*	Some are involved in the de novo synthesis of essential vitamins for the host, including vit. B12	[27,28,29,31,33,34,36]
*Fusobacteria*	<1%	*Fusobacterium*	β-lactamase production	[24,27,29,31,37]
*Verrucomicrobia*	<1%	*Akkermansia*	Some are involved in the production of SCFAs	[24,27,29,31,34]

**Table 2:** The influence of neurotransmitters and metabolites of gut microbiota on glioma.

	What Effect Does It Have?	References
**Neurotransmitters**		
GABA	Activation of the GABAA-R receptor induces cell depolarization through the efflux of chloride ions, thereby suppressing glioma cell proliferation and promoting cellular quiescence.	[72]
Serotonin	Activation of 5-HT1 and 5-HT2 receptors enhances cell proliferation, differentiation, migration, and gene expression in glioma cells. Specifically, activation of 5-HT2 receptors increases the expression of glial cell line-derived neurotrophic factor (GDNF) mRNA and the secretion of GDNF by C6 cells, which supports the survival, proliferation, and activation of glioma cells.	[73,74,75,76,77]
Glutamate	High levels of metabotropic glutamate receptor class II (GluR1/GluR4) contribute to increased cell proliferation and migration, as well as heightened activation of the MAPK and PI3K pathways. The release of glutamate further promotes the growth of malignant gliomas. Overexpression of calcium-permeable AMPA (α-amino-3-hydroxy-5-methyl-4-isoxazolepropionate) receptors facilitates tumor cell migration and proliferation by activating the PI3K/AKT signaling pathway. Additionally, AMPA receptor activation promotes perivascular invasion through β1-integrin-dependent adhesion to the extracellular matrix, both in vitro and in vivo.	[78,79,80,81,82,83,84]
Dopamine	It regulates cell survival and proliferation. The activity of the D2 receptor, in conjunction with the epidermal growth factor receptor (EGFR), is linked to increased proliferation of spheroids enriched with cancer stem cells.	[85,86,87]
Norepinephrine	Activation of the β2-adrenergic receptor inhibits the proliferation of astrocytoma 1321N1 cells. Norepinephrine suppresses MMP-11, thereby inhibiting the migration and invasion of glioblastoma cells. However, other studies have shown that β2-adrenergic receptor activation induces phosphorylation of extracellular signal-regulated kinases 1 and 2 (ERK1/2), which can increase the expression of matrix metalloproteinases (MMPs) and promote the proliferation of U251 glioblastoma cell lines.	[87,88,89]
**Gut microbiota metabolites**		
Tryptophan	Activates the aryl hydrocarbon receptor (AhR), which modulates the immune response and supports glioma cell survival. It also participates in the kynurenine pathway, contributing to immunosuppression and nicotinamide adenine dinucleotide (NAD+) metabolism, thereby promoting glioma growth.	[14,88,89,90]
Glutamine, glutamate	Increases neurotoxicity and supports glioma growth. Glutamate is metabolized into α-ketoglutarate (α-KG), linking it to the tricarboxylic acid (TCA) cycle. In gliomas, α-KG fuels the TCA cycle, maintains redox balance, and regulates epigenetic modifications essential for tumor proliferation and survival.	[91,92,93,94]
SCFA	Short-chain fatty acids (SCFAs) regulate inflammation and epigenetic pathways, shaping the glioma microenvironment and influencing its aggressiveness. They modulate the inflammatory cascade by inhibiting NF-κB and histone deacetylase pathways. A reduction in circulating SCFAs leads to a state of chronic stress, which influences tumor development through stress-related pathways. Butyrate induces Treg differentiation, while propionate inhibits glioma development and progression by promoting apoptosis and autophagy via peroxisome proliferator-activated receptor gamma (PPAR-γ) signaling, thereby counteracting tumorigenesis and slowing tumor growth. Both butyrate and propionate reduce VEGF levels and downregulate the PI3K/Akt/mTOR signaling pathway. In contrast, acetate affects acetyl-CoA production in glioma cells, leading to Rictor acetylation and the activation of mTORC2, which drives tumor proliferation.	[6,95,96,97,98,99]
LPSs	Lipopolysaccharides (LPSs) promote the migration and invasion of tumor cells by inducing the activation of the PI3K/Akt/mTOR pathway. LPSs can also over-activate Kirsten rat sarcoma virus (KRAS), contributing to carcinogenesis. Additionally, LPSs upregulate the expression of VEGFR by enhancing NF-κB activity, thereby promoting tumor angiogenesis.	[100,101]
Arginine	Arginine-derived metabolites in the body include polyamines and nitric oxide (NO). Polyamines promote the expression of ornithine decarboxylase, spermidine, spermine acetyltransferase, and serine/threonine kinase 1 (Akt1), driving tumor proliferation and metastasis. NO exhibits dual effects. It can induce tumor apoptosis through DNA and mitochondrial damage. However, elevated levels of NO inhibit NF-κB activity, which promotes angiogenesis and glioma growth. Additionally, NO acts as a factor that induces T cell apoptosis, contributing to immune suppression.	[102,103,104,105,106]
Bile acids (DCA, LCA, UDCA)	Deoxycholic acid (DCA) increases the activity of the VEGF and EGF pathways by activating their receptors and also stimulates the PI3K/Akt pathway. In contrast, ursodeoxycholic acid (UDCA) acts in the opposite way, partially inhibiting EGFR and promoting cancer cell apoptosis. UDCA contributes to decreased mitochondrial membrane potential, overproduction of reactive oxygen species (ROS), and endoplasmic reticulum stress. Lithocholic acid (LCA) activates the NF-κB pathway and induces ROS production, leading to oxidative DNA damage and inflammatory reactions.	[6,107]
TMAO	Trimethylamine N-oxide (TMAO) activates the NF-κB pathway and increases the production and secretion of VEGF from tumor cells, promoting angiogenesis and enhancing CD8+ T cell-dependent antitumor immunity.	[6]

## Data Availability

Available on request and with regulations.

## References

[1] Miranda-Filho A., Piñeros M., Soerjomataram I., Deltour I., Bray F. (2017). Cancers of the Brain and CNS: Global Patterns and Trends in Incidence. Neuro-Oncology.

[2] Cryan J.F., O’Riordan K.J., Cowan C.S.M., Sandhu K.V., Bastiaanssen T.F.S., Boehme M., Codagnone M.G., Cussotto S., Fulling C., Golubeva A.V. (2019). The Microbiota-Gut-Brain Axis. Physiol. Rev..

[3] Hagemeyer H., Hellwinkel O.J.C., Plata-Bello J. (2024). Zonulin as Gatekeeper in Gut–Brain Axis: Dysregulation in Glioblastoma. Biomedicines.

[4] Wikoff W.R., Anfora A.T., Liu J., Schultz P.G., Lesley S.A., Peters E.C., Siuzdak G. (2009). Metabolomics Analysis Reveals Large Effects of Gut Microflora on Mammalian Blood Metabolites. Proc. Natl. Acad. Sci. USA.

[5] Whitfield C., Trent M.S. (2014). Biosynthesis and Export of Bacterial Lipopolysaccharides. Annu. Rev. Biochem..

[6] Gong L., Yang S., Huang J., Li Y. (2024). Modulation of Gut Microbiota in Targeted Cancer Therapy: Insights on the EGFR/VEGF/KRAS Pathways. Cancer Biol. Med..

[7] Özoğul F. (2004). Production of Biogenic Amines by *Morganella morganii*, *Klebsiella pneumoniae* and *Hafnia alvei* Using a Rapid HPLC Method. Eur. Food Res. Technol..

[8] Barrett E., Ross R.P., O’Toole P.W., Fitzgerald G.F., Stanton C. (2012). γ-Aminobutyric Acid Production by Culturable Bacteria from the Human Intestine. J. Appl. Microbiol..

[9] Stanaszek P.M., Snell J.F., O’Neill J.J. (1977). Isolation, Extraction, and Measurement of Acetylcholine from Lactobacillus Plantarum. Appl. Environ. Microbiol..

[10] Landete J.M., de las Rivas B., Marcobal A., Muñoz R. (2007). Molecular Methods for the Detection of Biogenic Amine-Producing Bacteria on Foods. Int. J. Food Microbiol..

[11] Shishov V.A., Kirovskaya T.A., Kudrin V.S., Oleskin A.V. (2009). Amine Neuromediators, Their Precursors, and Oxidation Products in the Culture of Escherichia Coli K-12. Appl. Biochem. Microbiol..

[12] Komatsuzaki N., Shima J., Kawamoto S., Momose H., Kimura T. (2005). Production of γ-Aminobutyric Acid (GABA) by Lactobacillus Paracasei Isolated from Traditional Fermented Foods. Food Microbiol..

[13] Nwabo Kamdje A.H., Tagne Simo R., Fogang Dongmo H.P., Bidias A.R., Masumbe Netongo P. (2023). Role of Signaling Pathways in the Interaction between Microbial, Inflammation and Cancer. Holist. Integr. Oncol..

[14] Adams S., Braidy N., Bessesde A., Brew B.J., Grant R., Teo C., Guillemin G.J. (2012). The Kynurenine Pathway in Brain Tumor Pathogenesis. Cancer Res..

[15] Guillemin G.J. (2012). Quinolinic Acid, the Inescapable Neurotoxin. FEBS J..

[16] Farmanfarma K.h.K., Mohammadian M., Shahabinia Z., Hassanipour S., Salehiniya H. (2019). Brain Cancer in the World: An Epidemiological Review. World Cancer Res. J..

[17] Bray F., Laversanne M., Sung H., Ferlay J., Siegel R.L., Soerjomataram I., Jemal A. (2024). Global Cancer Statistics 2022: GLOBOCAN Estimates of Incidence and Mortality Worldwide for 36 Cancers in 185 Countries. CA Cancer J. Clin..

[18] CBTRUS CBTRUS Fact Sheet 2024. https://cbtrus.org/cbtrus-fact-sheet/.

[19] Patel A.P., Fisher J.L., Nichols E., Abd-Allah F., Abdela J., Abdelalim A., Abraha H.N., Agius D., Alahdab F., Alam T. (2019). Global, Regional, and National Burden of Brain and Other CNS Cancer, 1990–2016: A Systematic Analysis for the Global Burden of Disease Study 2016. Lancet Neurol..

[20] Ilic I., Ilic M. (2023). International Patterns and Trends in the Brain Cancer Incidence and Mortality: An Observational Study Based on the Global Burden of Disease. Heliyon.

[21] Yang K., Wu Z., Zhang H., Zhang N., Wu W., Wang Z., Dai Z., Zhang X., Zhang L., Peng Y. (2022). Glioma Targeted Therapy: Insight into Future of Molecular Approaches. Mol. Cancer.

[22] Esemen Y., Awan M., Parwez R., Baig A., Rahman S., Masala I., Franchini S., Giakoumettis D. (2022). Molecular Pathogenesis of Glioblastoma in Adults and Future Perspectives: A Systematic Review. Int. J. Mol. Sci..

[23] Packer R.J., MacDonald T., Vezina G. (2010). Central Nervous System Tumors. Hematol./Oncol. Clin. N. Am..

[24] Rosenbaum M., Knight R., Leibel R.L. (2015). The Gut Microbiota in Human Energy Homeostasis and Obesity. Trends Endocrinol. Metab..

[25] Altveş S., Yildiz H.K., Vural H.C. (2019). Interaction of the Microbiota with the Human Body in Health and Diseases. Biosci. Microbiota Food Health.

[26] Consortium T.H.M.P. (2012). Structure, Function and Diversity of the Healthy Human Microbiome. Nature.

[27] Rinninella E., Raoul P., Cintoni M., Franceschi F., Miggiano G.A.D., Gasbarrini A., Mele M.C. (2019). What Is the Healthy Gut Microbiota Composition? A Changing Ecosystem across Age, Environment, Diet, and Diseases. Microorganisms.

[28] Bibbò S., Lopetuso L.R., Ianiro G., Rienzo T.D., Gasbarrini A., Cammarota G. (2014). Role of Microbiota and Innate Immunity in Recurrent Clostridium Difficile Infection. J. Immunol. Res..

[29] Rinninella E., Cintoni M., Raoul P., Lopetuso L.R., Scaldaferri F., Pulcini G., Miggiano G.A.D., Gasbarrini A., Mele M.C. (2019). Food Components and Dietary Habits: Keys for a Healthy Gut Microbiota Composition. Nutrients.

[30] Mu C., Yang Y., Zhu W. (2016). Gut Microbiota: The Brain Peacekeeper. Front. Microbiol..

[31] Principi N., Esposito S. (2016). Gut Microbiota and Central Nervous System Development. J. Infect..

[32] Żakowicz J., Bramorska A., Zarzycka W., Kovbasiuk A., Kuć K., Brzezicka A. (2020). Wpływ mikrobiomu jelitowego na mózg i psychikę. Kosmos.

[33] Bibbò S., Ianiro G., Giorgio V., Scaldaferri F., Masucci L., Gasbarrini A., Cammarota G. (2016). The Role of Diet on Gut Microbiota Composition. Eur. Rev. Med. Pharmacol. Sci..

[34] Houtman T.A., Eckermann H.A., Smidt H., de Weerth C. (2022). Gut Microbiota and BMI throughout Childhood: The Role of Firmicutes, Bacteroidetes, and Short-Chain Fatty Acid Producers. Sci. Rep..

[35] Kumar S., Kumar A. (2022). Microbial Pathogenesis in Inflammatory Bowel Diseases. Microb. Pathog..

[36] Thursby E., Juge N. (2017). Introduction to the Human Gut Microbiota. Biochem. J..

[37] Brook I. (2015). Fusobacterial Head and Neck Infections in Children. Int. J. Pediatr. Otorhinolaryngol..

[38] Heijtz R.D., Wang S., Anuar F., Qian Y., Björkholm B., Samuelsson A., Hibberd M.L., Forssberg H., Pettersson S. (2011). Normal Gut Microbiota Modulates Brain Development and Behavior. Proc. Natl. Acad. Sci. USA.

[39] Bravo J.A., Forsythe P., Chew M.V., Escaravage E., Savignac H.M., Dinan T.G., Bienenstock J., Cryan J.F. (2011). Ingestion of Lactobacillus Strain Regulates Emotional Behavior and Central GABA Receptor Expression in a Mouse via the Vagus Nerve. Proc. Natl. Acad. Sci. USA.

[40] Stilling R.M., Ryan F.J., Hoban A.E., Shanahan F., Clarke G., Claesson M.J., Dinan T.G., Cryan J.F. (2015). Microbes & Neurodevelopment–Absence of Microbiota during Early Life Increases Activity-Related Transcriptional Pathways in the Amygdala. Brain Behav. Immun..

[41] Gao J., Xu K., Liu H., Liu G., Bai M., Peng C., Li T., Yin Y. (2018). Impact of the Gut Microbiota on Intestinal Immunity Mediated by Tryptophan Metabolism. Front. Cell. Infect. Microbiol..

[42] Kabouridis P.S., Lasrado R., McCallum S., Chng S.H., Snippert H.J., Clevers H., Pettersson S., Pachnis V. (2015). Microbiota Controls the Homeostasis of Glial Cells in the Gut Lamina Propria. Neuron.

[43] Clarke G., Stilling R.M., Kennedy P.J., Stanton C., Cryan J.F., Dinan T.G. (2014). Minireview: Gut Microbiota: The Neglected Endocrine Organ. Mol. Endocrinol..

[44] Mirzaei R., Bouzari B., Hosseini-Fard S.R., Mazaheri M., Ahmadyousefi Y., Abdi M., Jalalifar S., Karimitabar Z., Teimoori A., Keyvani H. (2021). Role of Microbiota-Derived Short-Chain Fatty Acids in Nervous System Disorders. Biomed. Pharmacother..

[45] Silva Y.P., Bernardi A., Frozza R.L. (2020). The Role of Short-Chain Fatty Acids From Gut Microbiota in Gut-Brain Communication. Front. Endocrinol..

[46] Jiang H., Zeng W., Zhang X., Pei Y., Zhang H., Li Y. (2022). The Role of Gut Microbiota in Patients with Benign and Malignant Brain Tumors: A Pilot Study. Bioengineered.

[47] Li Y., Jiang H., Wang X., Liu X., Huang Y., Wang Z., Ma Q., Dong L., Qi Y., Zhang H. (2022). Crosstalk Between the Gut and Brain: Importance of the Fecal Microbiota in Patient with Brain Tumors. Front. Cell. Infect. Microbiol..

[48] Yan J., Li B., Luo C. (2024). Gut Microbiota’s Role in Glioblastoma Risk, with a Focus on the Mediating Role of Metabolites. Front. Neurol..

[49] Wang S., Yin F., Guo Z., Li R., Sun W., Wang Y., Geng Y., Sun C., Sun D. (2024). Association between Gut Microbiota and Glioblastoma: A Mendelian Randomization Study. Front. Genet..

[50] Loh J.S., Mak W.Q., Tan L.K.S., Ng C.X., Chan H.H., Yeow S.H., Foo J.B., Ong Y.S., How C.W., Khaw K.Y. (2024). Microbiota–Gut–Brain Axis and Its Therapeutic Applications in Neurodegenerative Diseases. Signal Transduct. Target. Ther..

[51] Green G.B.H., Cox-Holmes A.N., Potier A.C.E., Marlow G.H., McFarland B.C. (2024). Modulation of the Immune Environment in Glioblastoma by the Gut Microbiota. Biomedicines.

[52] Larauche M., Mulak A., Taché Y. (2011). Stress and visceral pain: From animal models to clinical therapies. Exp. Neurol..

[53] Larauche M., Mulak A., Taché Y. (2011). Stress-Related Alterations of Visceral Sensation: Animal Models for Irritable Bowel Syndrome Study. J. Neurogastroenterol. Motil..

[54] Clarke G., Grenham S., Scully P., Fitzgerald P., Moloney R.D., Shanahan F., Dinan T.G., Cryan J.F. (2013). The Microbiome-Gut-Brain Axis during Early Life Regulates the Hippocampal Serotonergic System in a Sex-Dependent Manner. Mol. Psychiatry.

[55] Desbonnet L., Garrett L., Clarke G., Bienenstock J., Dinan T.G. (2008). The Probiotic Bifidobacteria Infantis: An Assessment of Potential Antidepressant Properties in the Rat. J. Psychiatr. Res..

[56] Longo S., Rizza S., Federici M. (2023). Microbiota-Gut-Brain Axis: Relationships among the Vagus Nerve, Gut Microbiota, Obesity, and Diabetes. Acta Diabetol..

[57] Sudo N., Chida Y., Aiba Y., Sonoda J., Oyama N., Yu X.-N., Kubo C., Koga Y. (2004). Postnatal Microbial Colonization Programs the Hypothalamic–Pituitary–Adrenal System for Stress Response in Mice. J. Physiol..

[58] Bonaz B., Bazin T., Pellissier S. (2018). The Vagus Nerve at the Interface of the Microbiota-Gut-Brain Axis. Front. Neurosci..

[59] Wang F.-B., Powley T.L. (2007). Vagal Innervation of Intestines: Afferent Pathways Mapped with New En Bloc Horseradish Peroxidase Adaptation. Cell Tissue Res..

[60] Furness J.B., Callaghan B.P., Rivera L.R., Cho H.-J., Lyte M., Cryan J.F. (2014). The Enteric Nervous System and Gastrointestinal Innervation: Integrated Local and Central Control. Microbial Endocrinology: The Microbiota-Gut-Brain Axis in Health and Disease.

[61] Furness J.B. (2008). The Enteric Nervous System.

[62] Li Y., Hao Y., Zhu J., Owyang C. (2000). Serotonin Released from Intestinal Enterochromaffin Cells Mediates Luminal Non–Cholecystokinin-Stimulated Pancreatic Secretion in Rats. Gastroenterology.

[63] Reigstad C.S., Salmonson C.E., Rainey J.F., Szurszewski J.H., Linden D.R., Sonnenburg J.L., Farrugia G., Kashyap P.C. (2014). Gut microbes promote colonic serotonin production through an effect of short-chain fatty acids on enterochromaffin cells. FASEB J..

[64] Mayer E.A., Tillisch K., Gupta A. (2015). Gut/Brain Axis and the Microbiota. J. Clin. Investig..

[65] Mitchell R.W., On N.H., Del Bigio M.R., Miller D.W., Hatch G.M. (2011). Fatty Acid Transport Protein Expression in Human Brain and Potential Role in Fatty Acid Transport across Human Brain Microvessel Endothelial Cells. J. Neurochem..

[66] Vijay N., Morris M.E. (2014). Role of Monocarboxylate Transporters in Drug Delivery to the Brain. Curr. Pharm. Des..

[67] Erny D., Hrabě de Angelis A.L., Jaitin D., Wieghofer P., Staszewski O., David E., Keren-Shaul H., Mahlakoiv T., Jakobshagen K., Buch T. (2015). Host Microbiota Constantly Control Maturation and Function of Microglia in the CNS. Nat. Neurosci..

[68] Nøhr M.K., Pedersen M.H., Gille A., Egerod K.L., Engelstoft M.S., Husted A.S., Sichlau R.M., Grunddal K.V., Seier Poulsen S., Han S. (2013). GPR41/FFAR3 and GPR43/FFAR2 as Cosensors for Short-Chain Fatty Acids in Enteroendocrine Cells vs FFAR3 in Enteric Neurons and FFAR2 in Enteric Leukocytes. Endocrinology.

[69] Tibbs T.N., Lopez L.R., Arthur J.C. (2019). The influence of the microbiota on immune development, chronic inflammation, and cancer in the context of aging. Microb. Cell.

[70] Zackular J.P., Baxter N.T., Iverson K.D., Sadler W.D., Petrosino J.F., Chen G.Y., Schloss P.D. (2013). The Gut Microbiome Modulates Colon Tumorigenesis. mBio.

[71] Tomkovich S., Dejea C.M., Winglee K., Drewes J.L., Chung L., Housseau F., Pope J.L., Gauthier J., Sun X., Mühlbauer M. (2019). Human Colon Mucosal Biofilms from Healthy or Colon Cancer Hosts Are Carcinogenic. J. Clin. Investig..

[72] Blanchart A., Fernando R., Häring M., Assaife-Lopes N., Romanov R.A., Andäng M., Harkany T., Ernfors P. (2017). Endogenous GABAA Receptor Activity Suppresses Glioma Growth. Oncogene.

[73] Merzak A., Koochekpour S., Fillion M.-P., Fillion G., Pilkington G.J. (1996). Expression of Serotonin Receptors in Human Fetal Astrocytes and Glioma Cell Lines: A Possible Role in Glioma Cell Proliferation and Migration. Mol. Brain Res..

[74] Siddiqui E.J., Thompson C.S., Mikhailidis D.P., Mumtaz F.H. (2005). The Role of Serotonin in Tumour Growth (Review). Oncol. Rep..

[75] Hisaoka K., Nishida A., Takebayashi M., Koda T., Yamawaki S., Nakata Y. (2004). Serotonin Increases Glial Cell Line-Derived Neurotrophic Factor Release in Rat C6 Glioblastoma Cells. Brain Res..

[76] Lu D.-Y., Leung Y.-M., Cheung C.-W., Chen Y.-R., Wong K.-L. (2010). Glial Cell Line-Derived Neurotrophic Factor Induces Cell Migration and Matrix Metalloproteinase-13 Expression in Glioma Cells. Biochem. Pharmacol..

[77] Wiesenhofer B., Stockhammer G., Kostron H., Maier H., Hinterhuber H., Humpel C. (2000). Glial Cell Line-Derived Neurotrophic Factor (GDNF) and Its Receptor (GFR-A1) Are Strongly Expressed in Human Gliomas. Acta Neuropathol..

[78] Pereira M.S.L., Klamt F., Thomé C.C., Worm P.V., Oliveira D.L. (2017). de Metabotropic Glutamate Receptors as a New Therapeutic Target for Malignant Gliomas. Oncotarget.

[79] Arcella A., Carpinelli G., Battaglia G., D’Onofrio M., Santoro F., Ngomba R.T., Bruno V., Casolini P., Giangaspero F., Nicoletti F. (2005). Pharmacological Blockade of Group II Metabotropic Glutamate Receptors Reduces the Growth of Glioma Cells in Vivo. Neuro-Oncology.

[80] Takano T., Lin J.H.-C., Arcuino G., Gao Q., Yang J., Nedergaard M. (2001). Glutamate Release Promotes Growth of Malignant Gliomas. Nat. Med..

[81] de Groot J., Sontheimer H. (2011). Glutamate and the biology of gliomas. Glia.

[82] Ishiuchi S., Tsuzuki K., Yoshida Y., Yamada N., Hagimura N., Okado H., Miwa A., Kurihara H., Nakazato Y., Tamura M. (2002). Blockage of Ca^2+^-Permeable AMPA Receptors Suppresses Migration and Induces Apoptosis in Human Glioblastoma Cells. Nat. Med..

[83] Piao Y., Lu L., de Groot J. (2009). AMPA Receptors Promote Perivascular Glioma Invasion via Β1 Integrin–Dependent Adhesion to the Extracellular Matrix. Neuro-Oncology.

[84] Lyons S.A., Chung W.J., Weaver A.K., Ogunrinu T., Sontheimer H. (2007). Autocrine Glutamate Signaling Promotes Glioma Cell Invasion. Cancer Res..

[85] Li J., Zhu S., Kozono D., Ng K., Futalan D., Shen Y., Akers J.C., Steed T., Kushwaha D., Schlabach M. (2014). Genome-Wide shRNA Screen Revealed Integrated Mitogenic Signaling between Dopamine Receptor D2 (DRD2) and Epidermal Growth Factor Receptor (EGFR) in Glioblastoma. Oncotarget.

[86] Weissenrieder J.S., Reed J.L., Green M.V., Moldovan G.-L., Koubek E.J., Neighbors J.D., Hohl R.J. (2019). The Dopamine D2 Receptor Contributes to the Spheroid Formation Behavior of U87 Glioblastoma Cells. Pharmacology.

[87] Bartek J., Hodny Z. (2014). Dopamine signaling: Target in glioblastoma. Oncotarget.

[88] Zelante T., Iannitti R.G., Cunha C., De Luca A., Giovannini G., Pieraccini G., Zecchi R., D’Angelo C., Massi-Benedetti C., Fallarino F. (2013). Tryptophan Catabolites from Microbiota Engage Aryl Hydrocarbon Receptor and Balance Mucosal Reactivity via Interleukin-22. Immunity.

[89] Gramatzki D., Pantazis G., Schittenhelm J., Tabatabai G., Köhle C., Wick W., Schwarz M., Weller M., Tritschler I. (2009). Aryl Hydrocarbon Receptor Inhibition Downregulates the TGF-Β/Smad Pathway in Human Glioblastoma Cells. Oncogene.

[90] Molfino A., Imbimbo G., Gallicchio C., Muscaritoli M. (2024). Tryptophan metabolism and kynurenine metabolites in cancer: Systemic nutritional and metabolic implications. Curr. Opin. Clin. Nutr. Metab. Care.

[91] Ye Z.-C., Rothstein J.D., Sontheimer H. (1999). Compromised Glutamate Transport in Human Glioma Cells: Reduction–Mislocalization of Sodium-Dependent Glutamate Transporters and Enhanced Activity of Cystine–Glutamate Exchange. J. Neurosci..

[92] Seltzer M.J., Bennett B.D., Joshi A.D., Gao P., Thomas A.G., Ferraris D.V., Tsukamoto T., Rojas C.J., Slusher B.S., Rabinowitz J.D. (2010). Inhibition of Glutaminase Preferentially Slows Growth of Glioma Cells with Mutant IDH1. Cancer Res..

[93] Dang L., White D.W., Gross S., Bennett B.D., Bittinger M.A., Driggers E.M., Fantin V.R., Jang H.G., Jin S., Keenan M.C. (2009). Cancer-Associated IDH1 Mutations Produce 2-Hydroxyglutarate. Nature.

[94] Maus A., Peters G.J. (2017). Glutamate and α-Ketoglutarate: Key Players in Glioma Metabolism. Amino Acids.

[95] Huuskonen J., Suuronen T., Nuutinen T., Kyrylenko S., Salminen A. (2004). Regulation of Microglial Inflammatory Response by Sodium Butyrate and Short-Chain Fatty Acids. Br. J. Pharmacol..

[96] Filippone A., Casili G., Scuderi S.A., Mannino D., Lanza M., Campolo M., Paterniti I., Capra A.P., Colarossi C., Bonasera A. (2023). Sodium Propionate Contributes to Tumor Cell Growth Inhibition through PPAR-γ Signaling. Cancers.

[97] Mashimo T., Pichumani K., Vemireddy V., Hatanpaa K.J., Singh D.K., Sirasanagandla S., Nannepaga S., Piccirillo S.G., Kovacs Z., Foong C. (2014). Acetate Is a Bioenergetic Substrate for Human Glioblastoma and Brain Metastases. Cell.

[98] Masui K., Tanaka K., Ikegami S., Villa G.R., Yang H., Yong W.H., Cloughesy T.F., Yamagata K., Arai N., Cavenee W.K. (2015). Glucose-Dependent Acetylation of Rictor Promotes Targeted Cancer Therapy Resistance. Proc. Natl. Acad. Sci. USA.

[99] Inan M.S., Rasoulpour R.J., Yin L., Hubbard A.K., Rosenberg D.W., Giardina C. (2000). The Luminal Short-Chain Fatty Acid Butyrate Modulates NF-κB Activity in a Human Colonic Epithelial Cell Line. Gastroenterology.

[100] Zhu G., Huang Q., Zheng W., Huang Y., Hua J., Yang S., Zhuang J., Wang J., Chang J., Xu J. (2016). LPS Upregulated VEGFR-3 Expression Promote Migration and Invasion in Colorectal Cancer via a Mechanism of Increased NF-κB Binding to the Promoter of VEGFR-3. Cell. Physiol. Biochem..

[101] Guo W., Zhang Y., Guo S., Mei Z., Liao H., Dong H., Wu K., Ye H., Zhang Y., Zhu Y. (2021). Tumor Microbiome Contributes to an Aggressive Phenotype in the Basal-like Subtype of Pancreatic Cancer. Commun. Biol..

[102] Dai F., Yu W., Song J., Li Q., Wang C., Xie S. (2017). Extracellular polyamines-induced proliferation and migration of cancer cells by ODC, SSAT, and Akt1-mediated pathway. Anti-Cancer Drugs.

[103] Bonavida B., Garban H. (2015). Nitric Oxide-Mediated Sensitization of Resistant Tumor Cells to Apoptosis by Chemo-Immunotherapeutics. Redox Biol..

[104] DiDonato J.A., Mercurio F., Karin M. (2012). NF-κB and the Link between Inflammation and Cancer. Immunol. Rev..

[105] Katsuyama K., Shichiri M., Marumo F., Hirata Y. (1998). NO Inhibits Cytokine-Induced iNOS Expression and NF-κB Activation by Interfering with Phosphorylation and Degradation of IκB-α. Arterioscler. Thromb. Vasc. Biol..

[106] Rivoltini L., Carrabba M., Huber V., Castelli C., Novellino L., Dalerba P., Mortarini R., Arancia G., Anichini A., Fais S. (2002). Immunity to Cancer: Attack and Escape in T Lymphocyte–Tumor Cell Interaction. Immunol. Rev..

[107] Yao Z., Zhang X., Zhao F., Wang S., Chen A., Huang B., Wang J., Li X. (2020). Ursodeoxycholic Acid Inhibits Glioblastoma Progression via Endoplasmic Reticulum Stress Related Apoptosis and Synergizes with the Proteasome Inhibitor Bortezomib. ACS Chem. Neurosci..

[108] McNulty N.P., Wu M., Erickson A.R., Pan C., Erickson B.K., Martens E.C., Pudlo N.A., Muegge B.D., Henrissat B., Hettich R.L. (2013). Effects of Diet on Resource Utilization by a Model Human Gut Microbiota Containing Bacteroides Cellulosilyticus WH2, a Symbiont with an Extensive Glycobiome. PLoS Biol..

[109] Robert C., Chassard C., Lawson P.A., Bernalier-Donadille A. (2007). *Bacteroides cellulosilyticus* Sp. Nov., a Cellulolytic Bacterium from the Human Gut Microbial Community. Int. J. Syst. Evol. Microbiol..

[110] Arnolds K.L., Yamada E., Neff C.P., Schneider J.M., Palmer B.E., Lozupone C.A. (2023). Disruption of Genes Encoding Putative Zwitterionic Capsular Polysaccharides of Diverse Intestinal Bacteroides Reduces the Induction of Host Anti-Inflammatory Factors. Microb. Ecol..

[111] Shandilya S., Kumar S., Kumar Jha N., Kumar Kesari K., Ruokolainen J. (2022). Interplay of Gut Microbiota and Oxidative Stress: Perspective on Neurodegeneration and Neuroprotection. J. Adv. Res..

[112] Kunst C., Schmid S., Michalski M., Tümen D., Buttenschön J., Müller M., Gülow K. (2023). The Influence of Gut Microbiota on Oxidative Stress and the Immune System. Biomedicines.

[113] Dumitrescu L., Popescu-Olaru I., Cozma L., Tulbă D., Hinescu M.E., Ceafalan L.C., Gherghiceanu M., Popescu B.O. (2018). Oxidative Stress and the Microbiota-Gut-Brain Axis. Oxidative Med. Cell. Longev..

[114] Sun Y., Wang X., Li L., Zhong C., Zhang Y., Yang X., Li M., Yang C. (2024). The Role of Gut Microbiota in Intestinal Disease: From an Oxidative Stress Perspective. Front. Microbiol..

[115] Li X., Wu C., Chen N., Gu H., Yen A., Cao L., Wang E., Wang L. (2016). PI3K/Akt/mTOR Signaling Pathway and Targeted Therapy for Glioblastoma. Oncotarget.

[116] Hong Y., Boiti A., Vallone D., Foulkes N.S. (2024). Reactive Oxygen Species Signaling and Oxidative Stress: Transcriptional Regulation and Evolution. Antioxidants.

[117] Liu H., Tang T. (2023). MAPK Signaling Pathway-Based Glioma Subtypes, Machine-Learning Risk Model, and Key Hub Proteins Identification. Sci. Rep..

[118] Sahm F., Oezen I., Opitz C.A., Radlwimmer B., von Deimling A., Ahrendt T., Adams S., Bode H.B., Guillemin G.J., Wick W. (2013). The Endogenous Tryptophan Metabolite and NAD+ Precursor Quinolinic Acid Confers Resistance of Gliomas to Oxidative Stress. Cancer Res..

[119] Morgan M.J., Liu Z. (2011). Crosstalk of Reactive Oxygen Species and NF-κB Signaling. Cell Res..

[120] Qu R., Zhang Y., Ma Y., Zhou X., Sun L., Jiang C., Zhang Z., Fu W. (2023). Role of the Gut Microbiota and Its Metabolites in Tumorigenesis or Development of Colorectal Cancer. Adv. Sci..

[121] Semenova N., Garashchenko N., Kolesnikov S., Darenskaya M., Kolesnikova L. (2024). Gut Microbiome Interactions with Oxidative Stress: Mechanisms and Consequences for Health. Pathophysiology.

[122] Olivier C., Oliver L., Lalier L., Vallette F.M. (2021). Drug Resistance in Glioblastoma: The Two Faces of Oxidative Stress. Front. Mol. Biosci..

[123] Sharma V., Joseph C., Ghosh S., Agarwal A., Mishra M.K., Sen E. (2007). Kaempferol Induces Apoptosis in Glioblastoma Cells through Oxidative Stress. Mol. Cancer Ther..

[124] Khan M., Yi F., Rasul A., Li T., Wang N., Gao H., Gao R., Ma T. (2012). Alantolactone Induces Apoptosis in Glioblastoma Cells via GSH Depletion, ROS Generation, and Mitochondrial Dysfunction. IUBMB Life.

[125] Festa M., Capasso A., D’Acunto C.W., Masullo M., Rossi A.G., Pizza C., Piacente S. (2011). Xanthohumol Induces Apoptosis in Human Malignant Glioblastoma Cells by Increasing Reactive Oxygen Species and Activating MAPK Pathways. J. Nat. Prod..

[126] Singer E., Judkins J., Salomonis N., Matlaf L., Soteropoulos P., McAllister S., Soroceanu L. (2015). Reactive Oxygen Species-Mediated Therapeutic Response and Resistance in Glioblastoma. Cell Death Dis..

[127] Zheng S.-X., Chen J.-P., Liang R.-S., Zhuang B.-B., Wang C.-H., Zhang G.-L., Shi S.-S., Chen J. (2024). *Schizophyllum commune* Fruiting Body Polysaccharides Inhibit Glioma by Mediating ARHI Regulation of PI3K/AKT Signalling Pathway. Int. J. Biol. Macromol..

[128] Zabolotneva A.A., Shatova O.P., Shegai P.V., Shestopalov A.V. (2023). Immunosuppression in the Tumor Microenvironment Mediated by Metabolites Derived from the Gut Microbiota. Oncol. Adv..

[129] Li Y., Wang X., Qi S., Gao L., Huang G., Ren Z., Li K., Peng Y., Yi G., Guo J. (2021). Spliceosome-Regulated RSRP1-Dependent NF-κB Activation Promotes the Glioblastoma Mesenchymal Phenotype. Neuro-Oncology.

[130] Puliyappadamba V.T., Hatanpaa K.J., Chakraborty S., Habib A.A. (2014). The Role of NF-κB in the Pathogenesis of Glioma. Mol. Cell. Oncol..

[131] Weaver K.D., Yeyeodu S., Cusack J.C., Baldwin A.S., Ewend M.G. (2003). Potentiation of Chemotherapeutic Agents Following Antagonism of Nuclear Factor Kappa B in Human Gliomas. J. Neuro-Oncol..

[132] Bonavia R., Inda M.M., Vandenberg S., Cheng S.-Y., Nagane M., Hadwiger P., Tan P., Sah D.W.Y., Cavenee W.K., Furnari F.B. (2012). EGFRvIII Promotes Glioma Angiogenesis and Growth through the NF-κB, Interleukin-8 Pathway. Oncogene.

[133] Wang C.-Y., Cusack J.C., Liu R., Baldwin A.S. (1999). Control of Inducible Chemoresistance: Enhanced Anti-Tumor Therapy through Increased Apoptosis by Inhibition of NF-κB. Nat. Med..

[134] Nagai S., Washiyama K., Kurimoto M., Takaku A., Endo S., Kumanishi T. (2002). Aberrant nuclear factor-κB activity and its participation in the growth of human malignant astrocytoma. J. Neurosurg..

[135] Westhoff M.-A., Zhou S., Nonnenmacher L., Karpel-Massler G., Jennewein C., Schneider M., Halatsch M.-E., Carragher N.O., Baumann B., Krause A. (2013). Inhibition of NF-κB Signaling Ablates the Invasive Phenotype of Glioblastoma. Mol. Cancer Res..

[136] Coupienne I., Bontems S., Dewaele M., Rubio N., Habraken Y., Fulda S., Agostinis P., Piette J. (2011). NF-kappaB Inhibition Improves the Sensitivity of Human Glioblastoma Cells to 5-Aminolevulinic Acid-Based Photodynamic Therapy. Biochem. Pharmacol..

[137] Jiang G., Zhang L., Wang J., Zhou H. (2016). Baicalein Induces the Apoptosis of U251 Glioblastoma Cell Lines via the NF-kB-P65-Mediated Mechanism. Anim. Cells Syst..

[138] Avci N.G., Ebrahimzadeh-Pustchi S., Akay Y.M., Esquenazi Y., Tandon N., Zhu J.-J., Akay M. (2020). NF-κB Inhibitor with Temozolomide Results in Significant Apoptosis in Glioblastoma via the NF-κB(P65) and Actin Cytoskeleton Regulatory Pathways. Sci. Rep..

[139] Spielbauer J., Glotfelty E.J., Sarlus H., Harris R.A., Diaz Heijtz R., Karlsson T.E. (2024). Bacterial Peptidoglycan Signalling in Microglia: Activation by MDP via the NF-κB/MAPK Pathway. Brain Behav. Immun..

[140] Krawczyk A., Stadler S.M., Strzalka-Mrozik B. (2024). Nanomedicines for Dry Eye Syndrome: Targeting Oxidative Stress with Modern Nanomaterial Strategies. Molecules.

[141] Marina-García N., Franchi L., Kim Y.-G., Hu Y., Smith D.E., Boons G.-J., Núñez G. (2009). Clathrin- and Dynamin-Dependent Endocytic Pathway Regulates Muramyl Dipeptide Internalization and NOD2 Activation1. J. Immunol..

[142] Kim J.-Y., Omori E., Matsumoto K., Núñez G., Ninomiya-Tsuji J. (2008). TAK1 Is a Central Mediator of NOD2 Signaling in Epidermal Cells. J. Biol. Chem..

[143] Strober W., Watanabe T. (2011). NOD2, an Intracellular Innate Immune Sensor Involved in Host Defense and Crohn’s Disease. Mucosal Immunol..

[144] Shabab T., Khanabdali R., Moghadamtousi S.Z., Kadir H.A., Mohan G. (2017). Neuroinflammation Pathways: A General Review. Int. J. Neurosci..

[145] Avery T.Y., Köhler N., Zeiser R., Brummer T., Ruess D.A. (2022). Onco-Immunomodulatory Properties of Pharmacological Interference with RAS-RAF-MEK-ERK Pathway Hyperactivation. Front. Oncol..

[146] Nguyen K., Tran M.N., Rivera A., Cheng T., Windsor G.O., Chabot A.B., Cavanaugh J.E., Collins-Burow B.M., Lee S.B., Drewry D.H. (2022). MAP3K Family Review and Correlations with Patient Survival Outcomes in Various Cancer Types. Front. Biosci.-Landmark.

[147] Cuarental L., Sucunza-Sáenz D., Valiño-Rivas L., Fernandez-Fernandez B., Sanz A.B., Ortiz A., Vaquero J.J., Sanchez-Niño M.D. (2019). MAP3K Kinases and Kidney Injury. Nefrología.

[148] Campbell B.B., Galati M.A., Stone S.C., Riemenschneider A.N., Edwards M., Sudhaman S., Siddaway R., Komosa M., Nunes N.M., Nobre L. (2021). Mutations in the RAS/MAPK Pathway Drive Replication Repair–Deficient Hypermutated Tumors and Confer Sensitivity to MEK Inhibition. Cancer Discov..

[149] Kim E.K., Choi E.-J. (2010). Pathological Roles of MAPK Signaling Pathways in Human Diseases. Biochim. Biophys. Acta (BBA)-Mol. Basis Dis..

[150] Nigam M., Devi K., Coutinho H.D.M., Mishra A.P. (2024). Exploration of Gut Microbiome and Inflammation: A Review on Key Signalling Pathways. Cell. Signal..

[151] Barona I., Fagundes D.S., Gonzalo S., Grasa L., Arruebo M.P., Plaza M.Á., Murillo M.D. (2011). Role of TLR4 and MAPK in the Local Effect of LPS on Intestinal Contractility. J. Pharm. Pharmacol..

[152] Cantó E., Moga E., Ricart E., Garcia-Bosch O., Garcia-Planella E., Juarez C., Vidal S. (2009). MDP-Induced Selective Tolerance to TLR4 Ligands: Impairment in NOD2 Mutant Crohn’s Disease Patients. Inflamm. Bowel Dis..

[153] Cargnello M., Roux P.P. (2011). Activation and Function of the MAPKs and Their Substrates, the MAPK-Activated Protein Kinases. Microbiol. Mol. Biol. Rev..

[154] Gille H., Kortenjann M., Thomae O., Moomaw C., Slaughter C., Cobb M., Shaw P. (1995). ERK Phosphorylation Potentiates Elk-1-mediated Ternary Complex Formation and Transactivation. EMBO J..

[155] Trejo-Solís C., Castillo-Rodríguez R.A., Serrano-García N., Silva-Adaya D., Vargas-Cruz S., Chávez-Cortéz E.G., Gallardo-Pérez J.C., Zavala-Vega S., Cruz-Salgado A., Magaña-Maldonado R. (2024). Metabolic Roles of HIF1, c-Myc, and P53 in Glioma Cells. Metabolites.

[156] Liu Y.-S., Hsu J.-W., Lin H.-Y., Lai S.-W., Huang B.-R., Tsai C.-F., Lu D.-Y. (2019). Bradykinin B1 Receptor Contributes to Interleukin-8 Production and Glioblastoma Migration through Interaction of STAT3 and SP-1. Neuropharmacology.

[157] Abou-Ghazal M., Yang D.S., Qiao W., Reina-Ortiz C., Wei J., Kong L.-Y., Fuller G.N., Hiraoka N., Priebe W., Sawaya R. (2008). The Incidence, Correlation with Tumor-Infiltrating Inflammation, and Prognosis of Phosphorylated STAT3 Expression in Human Gliomas. Clin. Cancer Res..

[158] Wei Z., Jiang X., Qiao H., Zhai B., Zhang L., Zhang Q., Wu Y., Jiang H., Sun X. (2013). STAT3 Interacts with Skp2/P27/P21 Pathway to Regulate the Motility and Invasion of Gastric Cancer Cells. Cell. Signal..

[159] Liu Y.-S., Lin H.-Y., Lai S.-W., Huang C.-Y., Huang B.-R., Chen P.-Y., Wei K.-C., Lu D.-Y. (2017). MiR-181b Modulates EGFR-Dependent VCAM-1 Expression and Monocyte Adhesion in Glioblastoma. Oncogene.

[160] Mehdizadeh R., Madjid Ansari A., Forouzesh F., Shahriari F., Shariatpanahi S.P., Salaritabar A., Javidi M.A. (2023). P53 Status, and G2/M Cell Cycle Arrest, Are Determining Factors in Cell-Death Induction Mediated by ELF-EMF in Glioblastoma. Sci. Rep..

[161] Watson S.A., McStay G.P. (2020). Functions of Cytochrome c Oxidase Assembly Factors. Int. J. Mol. Sci..

[162] Vousden K.H., Ryan K.M. (2009). P53 and Metabolism. Nat. Rev. Cancer.

[163] Kawauchi K., Araki K., Tobiume K., Tanaka N. (2008). P53 Regulates Glucose Metabolism through an IKK-NF-κB Pathway and Inhibits Cell Transformation. Nat. Cell Biol..

[164] Li S., Zhang W., Chen B., Jiang T., Wang Z. (2010). Prognostic and Predictive Value of P53 in Low MGMT Expressing Glioblastoma Treated with Surgery, Radiation and Adjuvant Temozolomide Chemotherapy. Neurol. Res..

[165] Lyu Y., Yang H., Chen L. (2022). Metabolic Regulation on the Immune Environment of Glioma through Gut Microbiota. Semin. Cancer Biol..

[166] Vanhaesebroeck B., Guillermet-Guibert J., Graupera M., Bilanges B. (2010). The Emerging Mechanisms of Isoform-Specific PI3K Signalling. Nat. Rev. Mol. Cell Biol..

[167] Jakubowicz-Gil J. (2009). Inhibitory szlaku PI3K-Akt/PKB-mTOR w leczeniu glejaków. Postępy Biol. Komórki.

[168] Fingar D.C., Richardson C.J., Tee A.R., Cheatham L., Tsou C., Blenis J. (2004). mTOR Controls Cell Cycle Progression through Its Cell Growth Effectors S6K1 and 4E-BP1/Eukaryotic Translation Initiation Factor 4E. Mol. Cell. Biol..

[169] Yang M., Lu Y., Piao W., Jin H. (2022). The Translational Regulation in mTOR Pathway. Biomolecules.

[170] Rabanal-Ruiz Y., Otten E.G., Korolchuk V.I. (2017). mTORC1 as the Main Gateway to Autophagy. Essays Biochem..

[171] Kulkarni S., Goel-Bhattacharya S., Sengupta S., Cochran B.H. (2018). A Large-Scale RNAi Screen Identifies SGK1 as a Key Survival Kinase for GBM Stem Cells. Mol. Cancer Res..

[172] do Carmo A., Balça-Silva J., Matias D., Lopes M.C. (2013). PKC Signaling in Glioblastoma. Cancer Biol. Ther..

[173] Dehhaghi M., Kazemi Shariat Panahi H., Heng B., Guillemin G.J. (2020). The Gut Microbiota, Kynurenine Pathway, and Immune System Interaction in the Development of Brain Cancer. Front. Cell Dev. Biol..

[174] Grishanova A.Y., Perepechaeva M.L. (2024). Kynurenic Acid/AhR Signaling at the Junction of Inflammation and Cardiovascular Diseases. Int. J. Mol. Sci..

[175] Guillemin G.J., Cullen K.M., Lim C.K., Smythe G.A., Garner B., Kapoor V., Takikawa O., Brew B.J. (2007). Characterization of the Kynurenine Pathway in Human Neurons. J. Neurosci..

[176] Reyes Ocampo J., Lugo Huitrón R., González-Esquivel D., Ugalde-Muñiz P., Jiménez-Anguiano A., Pineda B., Pedraza-Chaverri J., Ríos C., Pérez de la Cruz V. (2014). Kynurenines with Neuroactive and Redox Properties: Relevance to Aging and Brain Diseases. Oxidative Med. Cell. Longev..

[177] Beninger R.J., Colton A.M., Ingles J.L., Jhamandas K., Boegman R.J. (1994). Picolinic Acid Blocks the Neurotoxic but Not the Neuroexcitant Properties of Quinolinic Acid in the Rat Brain: Evidence from Turning Behaviour and Tyrosine Hydroxylase Immunohistochemistry. Neuroscience.

[178] Sun T., Xie R., He H., Xie Q., Zhao X., Kang G., Cheng C., Yin W., Cong J., Li J. (2022). Kynurenic Acid Ameliorates NLRP3 Inflammasome Activation by Blocking Calcium Mobilization via GPR35. Front. Immunol..

[179] Du L., Xing Z., Tao B., Li T., Yang D., Li W., Zheng Y., Kuang C., Yang Q. (2020). Both IDO1 and TDO Contribute to the Malignancy of Gliomas via the Kyn–AhR–AQP4 Signaling Pathway. Signal Transduct. Target. Ther..

[180] Ishaq H.M., Yasin R., Mohammad I.S., Fan Y., Li H., Shahzad M., Xu J. (2024). The Gut-Brain-Axis: A Positive Relationship between Gut Microbial Dysbiosis and Glioblastoma Brain Tumour. Heliyon.

[181] Ting N.L.-N., Lau H.C.-H., Yu J. (2022). Cancer Pharmacomicrobiomics: Targeting Microbiota to Optimise Cancer Therapy Outcomes. Gut.

[182] Villéger R., Lopès A., Carrier G., Veziant J., Billard E., Barnich N., Gagnière J., Vazeille E., Bonnet M. (2019). Intestinal Microbiota: A Novel Target to Improve Anti-Tumor Treatment?. Int. J. Mol. Sci..

[183] Yan J., Yang L., Ren Q., Zhu C., Du H., Wang Z., Qi Y., Xian X., Chen D. (2024). Gut Microbiota as a Biomarker and Modulator of Anti-Tumor Immunotherapy Outcomes. Front. Immunol..

[184] Wang L., Li S., Fan H., Han M., Xie J., Du J., Peng F. (2022). *Bifidobacterium lactis* Combined with *Lactobacillus plantarum* Inhibit Glioma Growth in Mice through Modulating PI3K/AKT Pathway and Gut Microbiota. Front. Microbiol..

[185] Fan H., Wang Y., Han M., Wang L., Li X., Kuang X., Du J., Peng F. (2024). Multi-Omics-Based Investigation of Bifidobacterium’s Inhibitory Effect on Glioma: Regulation of Tumor and Gut Microbiota, and MEK/ERK Cascade. Front. Microbiol..

[186] De Cecco L., Biassoni V., Schiavello E., Carenzo A., Iannò M.F., Licata A., Marra M., Carollo M., Boschetti L., Massimino M. (2022). DIPG-36. The Brain-Gut-Microbiota Axis to Predict Outcome in Pediatric Diffuse Intrinsic Pontine Glioma. Neuro-Oncology.

[187] Fan Y., Su Q., Chen J., Wang Y., He S. (2022). Gut Microbiome Alterations Affect Glioma Development and Foxp3 Expression in Tumor Microenvironment in Mice. Front. Oncol..

[188] Wang W., Ou Z., Huang X., Wang J., Li Q., Wen M., Zheng L. (2024). Microbiota and Glioma: A New Perspective from Association to Clinical Translation. Gut Microbes.

[189] Zhang H., Hong Y., Wu T., Ben E., Li S., Hu L., Xie T. (2024). Role of Gut Microbiota in Regulating Immune Checkpoint Inhibitor Therapy for Glioblastoma. Front. Immunol..

[190] Reardon D., Idbaih A., Vieito M., Tabatabai G., Stradella A., Ghiringhelli F., Burger M., Mildenberger I., Herrlinger U., González M. (2022). 642 EO2401 Microbiome Derived Therapeutic Vaccine + Nivolumab, with/without Standard Continuous, or Low-Dose Symptom Directed, Bevacizumab, in Recurrent Glioblastoma: Phase 1–2 EOGBM1–18/ROSALIE Study. J. Immunother. Cancer.

[191] Dono A., Patrizz A., McCormack R.M., Putluri N., Ganesh B.P., Kaur B., McCullough L.D., Ballester Y.L., Esquenazi Y. (2020). Glioma Induced Alterations in Fecal Short-Chain Fatty Acids and Neurotransmitters. CNS Oncol..

[192] D’Alessandro G., Antonangeli F., Marrocco F., Porzia A., Lauro C., Santoni A., Limatola C. (2020). Gut Microbiota Alterations Affect Glioma Growth and Innate Immune Cells Involved in Tumor Immunosurveillance in Mice. Eur. J. Immunol..

[193] Wang J., Liu Y., Zhang A., Yu W., Lei Q., Xiao B., Luo Z. (2022). Investigational Microbiological Therapy for Glioma. Cancers.

[194] Vannini E., Maltese F., Olimpico F., Fabbri A., Costa M., Caleo M., Baroncelli L. (2017). Progression of Motor Deficits in Glioma-Bearing Mice: Impact of CNF1 Therapy at Symptomatic Stages. Oncotarget.

[195] Vannini E., Panighini A., Cerri C., Fabbri A., Lisi S., Pracucci E., Benedetto N., Vannozzi R., Fiorentini C., Caleo M. (2014). The Bacterial Protein Toxin, Cytotoxic Necrotizing Factor 1 (CNF1) Provides Long-Term Survival in a Murine Glioma Model. BMC Cancer.

[196] Naghavian R., Faigle W., Oldrati P., Wang J., Toussaint N.C., Qiu Y., Medici G., Wacker M., Freudenmann L.K., Bonté P.-E. (2023). Microbial Peptides Activate Tumour-Infiltrating Lymphocytes in Glioblastoma. Nature.

[197] Stupp R., Mason W.P., van den Bent M.J., Weller M., Fisher B., Taphoorn M.J.B., Belanger K., Brandes A.A., Marosi C., Bogdahn U. (2005). Radiotherapy plus Concomitant and Adjuvant Temozolomide for Glioblastoma. N. Engl. J. Med..

[198] Lu L., Li F., Gao Y., Kang S., Li J., Guo J. (2024). Microbiome in Radiotherapy: An Emerging Approach to Enhance Treatment Efficacy and Reduce Tissue Injury. Mol. Med..

[199] Çakır T., Güven M., Taşpınar M., Denizler F.N., Kartal B. (2019). The Effect of Sodium Butyrate on Radiosensitivity in Glioblastoma Cell. Van Med. J..

[200] Entin-Meer M., Rephaeli A., Yang X., Nudelman A., VandenBerg S.R., Haas-Kogan D.A. (2005). Butyric Acid Prodrugs Are Histone Deacetylase Inhibitors That Show Antineoplastic Activity and Radiosensitizing Capacity in the Treatment of Malignant Gliomas. Mol. Cancer Ther..

[201] Ito N., Sawa H., Nagane M., Noguchi A., Hara M., Saito I. (2001). Inhibitory Effects of Sodium Butyrate on Proliferation and Invasiveness of Human Glioma Cells. Neurosurgery.

[202] Okumura S., Konishi Y., Narukawa M., Sugiura Y., Yoshimoto S., Arai Y., Sato S., Yoshida Y., Tsuji S., Uemura K. (2021). Gut Bacteria Identified in Colorectal Cancer Patients Promote Tumourigenesis via Butyrate Secretion. Nat. Commun..

[203] Chen S.-M., Chieng W.-W., Huang S.-W., Hsu L.-J., Jan M.-S. (2020). The Synergistic Tumor Growth-Inhibitory Effect of Probiotic Lactobacillus on Transgenic Mouse Model of Pancreatic Cancer Treated with Gemcitabine. Sci. Rep..

[204] Ni Y., Li R., Shen X., Yi D., Ren Y., Wang F., Geng Y., You Q. (2024). *Diaphorobacter nitroreducens* Synergize with Oxaliplatin to Reduce Tumor Burden in Mice with Lung Adenocarcinoma. mSystems.

[205] Hou X., Du H., Deng Y., Wang H., Liu J., Qiao J., Liu W., Shu X., Sun B., Liu Y. (2023). Gut Microbiota Mediated the Individualized Efficacy of Temozolomide via Immunomodulation in Glioma. J. Transl. Med..

[206] Meléndez-Vázquez N.M., Nguyen T.T., Fan X., López-Rivas A.R., Fueyo J., Gomez-Manzano C., Godoy-Vitorino F. (2024). Gut Microbiota Composition Is Associated with the Efficacy of Delta-24-RGDOX in Malignant Gliomas. Mol. Ther. Oncol..

[207] Sun R., Liu M., Lu J., Chu B., Yang Y., Song B., Wang H., He Y. (2022). Bacteria Loaded with Glucose Polymer and Photosensitive ICG Silicon-Nanoparticles for Glioblastoma Photothermal Immunotherapy. Nat. Commun..

[208] Hao R., Liu Q., Wang L., Jian W., Cheng Y., Zhang Q., Hayer K., Kamarudin Raja Idris R., Zhang Y., Lu O. (2023). Anti-Inflammatory Effect of Lactiplantibacillus Plantarum T1 Cell-Free Supernatants through Suppression of Oxidative Stress and NF-κB- and MAPK-Signaling Pathways. Appl. Environ. Microbiol..

[209] Kim H.G., Kim N.-R., Gim M.G., Lee J.M., Lee S.Y., Ko M.Y., Kim J.Y., Han S.H., Chung D.K. (2008). Lipoteichoic Acid Isolated from Lactobacillus Plantarum Inhibits Lipopolysaccharide-Induced TNF-α Production in THP-1 Cells and Endotoxin Shock in Mice1. J. Immunol..

[210] Sordillo P.P., Sordillo L.A., Helson L. (2017). The Kynurenine Pathway: A Primary Resistance Mechanism in Patients with Glioblastoma. Anticancer Res..

[211] Song X., Si Q., Qi R., Liu W., Li M., Guo M., Wei L., Yao Z. (2021). Indoleamine 2,3-Dioxygenase 1: A Promising Therapeutic Target in Malignant Tumor. Front. Immunol..

[212] Zhai L., Lauing K.L., Chang A.L., Dey M., Qian J., Cheng Y., Lesniak M.S., Wainwright D.A. (2015). The Role of IDO in Brain Tumor Immunotherapy. J. Neurooncol..

[213] Pilotte L., Larrieu P., Stroobant V., Colau D., Dolušić E., Frédérick R., De Plaen E., Uyttenhove C., Wouters J., Masereel B. (2012). Reversal of Tumoral Immune Resistance by Inhibition of Tryptophan 2,3-Dioxygenase. Proc. Natl. Acad. Sci. USA.

[214] Ruiz-Rodado V., Dowdy T., Lita A., Kramp T., Zhang M., Jung J., Dios-Esponera A., Zhang L., Herold-Mende C.C., Camphausen K. (2022). Cysteine Is a Limiting Factor for Glioma Proliferation and Survival. Mol. Oncol..

[215] Liu H., Zhang W., Wang K., Wang X., Yin F., Li C., Wang C., Zhao B., Zhong C., Zhang J. (2015). Methionine and Cystine Double Deprivation Stress Suppresses Glioma Proliferation via Inducing ROS/Autophagy. Toxicol. Lett..

[216] Silver D.J., Lathia J.D., Hine C. (2021). Hydrogen Sulfide Operates as a Glioblastoma Suppressor and Is Lost under High Fat Diet. Mol. Cell. Oncol..

[217] Abdelwahab M.G., Fenton K.E., Preul M.C., Rho J.M., Lynch A., Stafford P., Scheck A.C. (2012). The Ketogenic Diet Is an Effective Adjuvant to Radiation Therapy for the Treatment of Malignant Glioma. PLoS ONE.

[218] Martuscello R.T., Vedam-Mai V., McCarthy D.J., Schmoll M.E., Jundi M.A., Louviere C.D., Griffith B.G., Skinner C.L., Suslov O., Deleyrolle L.P. (2016). A Supplemented High-Fat Low-Carbohydrate Diet for the Treatment of Glioblastoma. Clin. Cancer Res..

